# 14-3-3γ Knockdown promotes matrix mineralization in human mesenchymal stromal cells

**DOI:** 10.1038/s41419-026-08540-4

**Published:** 2026-03-27

**Authors:** Lautaro Rivera, Sergio Müller, Marina Uhart, Diego Martin Bustos

**Affiliations:** 1 Instituto de Histología y Embriología de Mendoza (IHEM, Universidad Nacional de Cuyo, CONICET), Mendoza, Argentina; 2 Facultad de Ciencias Exactas y Naturales, Universidad Nacional de Cuyo, Mendoza, Argentina

**Keywords:** Cell biology, Stem cells

## Abstract

The 14-3-3 proteins are emerging as important modulators of osteoblast differentiation and function. Recent studies highlight specific roles of 14-3-3 paralogs in bone physiology, with their dysregulation linked to impaired skeletal homeostasis and bone-related diseases. Among these, the 14-3-3γ paralog has been implicated in bone formation, though its precise role remains unclear.

In this study, we investigated the function of 14-3-3γ in the osteogenic differentiation of human adipose-derived mesenchymal stem/stromal cells (hASCs). Using an adenoviral system, we knocked down 14-3-3γ and assessed osteogenic markers. Tissue-Nonspecific Alkaline Phosphatase (TNAP) activity, RUNX2 protein levels, and the expression of osteogenic genes (BGLAP, SPP1) were analyzed during matrix maturation and mineralization. Calcium and collagen deposition were evaluated via Alizarin Red S and Aniline Blue staining, respectively, and compared with cells overexpressing recombinant 14-3-3γ. Proteomic profiling via quantitative mass spectrometry was performed to identify protein changes after 14-3-3γ silencing. Subcellular localization of endogenous 14-3-3γ was also examined during differentiation. Knockdown of 14-3-3γ enhanced TNAP activity and increased matrix mineralization, while its overexpression suppressed these processes. Proteomic analysis revealed enrichment of proteins related to endoplasmic reticulum stress and bone development. Furthermore, 14-3-3γ shifted from a diffuse to a peri-endoplasmic reticulum distribution, with increased colocalization with calnexin during osteogenic induction. These findings reveal a novel inhibitory role of 14-3-3γ in matrix mineralization of hASCs, suggesting that targeting this paralog may offer new avenues for therapies in bone remodeling disorders.

## Introduction

Bone is in a continuous state of remodeling through the coordinated actions of osteoblasts and osteoclasts, which mediate bone formation and resorption, respectively [1, 2]. Osteoblasts originate from the osteogenic differentiation of mesenchymal stem/stromal cells (MSCs), a process that comprises three main stages: proliferation, extracellular matrix (ECM) maturation, and ECM mineralization [3–5]. The early phase of commitment is marked by the activation of the Runt-related transcription factor 2 (RUNX2), known as a master regulator of osteogenesis, promoting the expression of bone matrix proteins while repressing adipogenesis and chondrogenesis [6]. As differentiation progresses, cell proliferation declines and expression of the early marker tissue-nonspecific alkaline phosphatase (TNAP) increases. TNAP plays a central role in mineralization by generating inorganic phosphate required for hydroxyapatite formation [7, 8]. Phenotypic changes include expansion of the endoplasmic reticulum (ER), enlarged nucleus, and an active Golgi apparatus, reflecting the elevated production and secretion of type I collagen and other non-collagenous ECM proteins (such as osteocalcin, osteopontin and osteonectin) during the maturation phase [9–11]. Matrix mineralization is subsequently achieved through the nucleation and deposition of hydroxyapatite crystals on the ECM, a process that requires a well-organized collagen type I scaffold, the enzymatic activity of TNAP, and the coordinated function of non-collagenous proteins [9, 10].

An increasing attention has been directed toward the role of 14-3-3 proteins, as additional modulators that influence osteoblast differentiation and function [12]. The 14-3-3 proteins (also known as tyrosine 3-monooxygenase/tryptophan 5-monooxygenase activation protein) represent a family of regulatory molecules involved in many cellular processes through protein-protein interactions [13]. The 14-3-3 association can control the function of the target protein, by influencing either its activity, stability, subcellular localization, or molecular interactions [14–16]. In mammals, there are seven highly conserved 14-3-3 paralogs (α/β, γ, ε, η, θ/τ, ζ, and σ), with over 600 reported binding partners [17]. Even though the family of 14-3-3 proteins shows functional redundancy, there is also evidence that indicates evolutionary and biochemical diversity, like subcellular localization, tissue-specific expression, target specificity, dimerization preferences (homo- or heterodimerization), and regulation through post-translational modifications [18–21].

Recent studies have begun to uncover the specific roles of individual 14-3-3 paralogs in bone physiology, highlighting how their dysregulation disrupt skeletal homeostasis and contribute to the development of bone pathologies [12]. Our recent study identified a distinct regulation pattern of 14-3-3 paralogs during the late-stage of osteogenic differentiation of human adipose-derived mesenchymal stem/stromal cells (hASCs), with the 14-3-3β and ε paralogs upregulated, and 14-3-3γ downregulated after 21 days post-induction [22]. Further investigation has revealed that 14-3-3β acts as a negative regulator of osteogenesis, while 14-3-3ε appears to promote it [23–25]. In contrast, what we know about 14-3-3γ is largely based upon indirect evidence [26, 27]. Data from proteomic analysis of extensively subcultured human mesenchymal stem cells demonstrated a correlation between reduced osteogenic potential and increased 14-3-3γ expression [26]. Overall, these insights associate the 14-3-3γ paralog with numerous processes relevant to bone formation; however, its role and mechanisms remain elusive.

Here, we studied the role of 14-3-3γ in the osteogenic differentiation of hASCs. To address this, we characterized the changes associated with the knockdown of 14-3-3γ during the ECM maturation and mineralization phases. In addition, we conducted the first proteomic analysis to identify alterations in protein abundance resulting from 14-3-3γ downregulation during late-stage osteogenesis, integrating bioinformatic and cell-based experimental approaches. Our data demonstrate for the first time that 14-3-3γ is a critical mediator of efficient osteogenic differentiation and reveal the mechanisms by which it negatively regulates matrix mineralization.

## Materials and methods

### Human adipose-derived mesenchymal stromal cells (hASCs) Isolation, culture and characterization

Subcutaneous adipose tissue was obtained from healthy female donors aged 29-45 years undergoing elective abdominal dermolipectomy. Written informed consent was obtained from all patients and approved by the Bioethics Committee at Universidad Nacional de Cuyo (EXP-FCM:14594/2014), in accordance with the Declaration of Helsinki.

Human Adipose-derived Mesenchymal Stromal Cells (hASCs) were isolated using an explant culture method [28, 29]. Briefly, adipose tissue was mechanically processed in small pieces of 1–3 mm³. Explants were distributed in a 10 cm culture dish, left for 5 min and then cover by Dulbecco’s modified Eagle’s medium (DMEM, Thermo Fisher Scientific) supplemented with 10% fetal bovine serum (FBS; Internegocios), 1% L-Glutamine, and antibiotics penicillin 10 U/mL and streptomycin 100 μg/mL (Thermo Fisher Scientific). Explants were removed on day 12. Cells were expanded upon reaching 90% of confluence. Surface marker characterization by flow cytometry and differentiation potential analysis were performed by following the standards established by the International Society for Cellular Therapy and the International Federation for Adipose Therapeutics and Science [30, 31], as shown in the Supplementary Data (Fig. S1).

Cells were characterized by flow cytometry using the BD Stemflow human MSC analysis kit (562245, BD Biosciences), which identifies positive surface markers (CD90, CD73, and CD105) and negative markers (CD34, CD19, CD45, CD11b, and HLA-DR). To evaluate the differentiation potential of hASCs into mature adipocytes, chondroblasts, and osteoblasts, cells at passages 1–2 were seeded at 4×10⁴ cells/cm² and cultured to 70–75% of confluence. At this point, the medium was replaced with lineage-specific differentiation media.

For adipogenesis, cells were cultured in high-glucose DMEM containing 10% FBS, 2.5 μM dexamethasone, 0.5 mM 3-isobutyl-1-methylxanthine (IBMX), and 10 µg/mL insulin for 10 days, with medium changes every 2–3 days. Adipogenic commitment was confirmed by Oil Red O staining of lipid droplets, following the protocol described by Gojanovich et al. [31].

For chondrogenesis, cells were centrifuged at 680 *g* for 5 min to form pellets, which were cultured for 21 days in a commercial chondrogenic medium (StemPro Chondrocyte Differentiation Kit, Thermo Fisher Scientific), with medium changes every 2–3 days. Pellets were then fixed, embedded in paraffin, and stained with Alcian Blue to detect proteoglycan-rich cartilaginous matrix, following the protocol described by Nam et al. [32].

For osteogenesis, hASCs were cultured for 21 days in low-glucose DMEM supplemented with 10% FBS, 0.1 µM dexamethasone, 50 µg/mL ascorbate-2-phosphate, and 10 mM β-glycerophosphate, with medium changes every 2–3 days. Extracellular matrix mineralization was evaluated by Alizarin Red S staining as detailed below.

### Construction of adenoviral vectors and virus production

For the knockdown of 14-3-3γ, an expression cassette for shRNA targeting 14-3-3γ under the control of a U6 promoter, previously cloned into a pENTR™ vector (Invitrogen), was transferred into the pAd/BLOCK-iT™-DEST adenoviral expression vector (Invitrogen) using the Gateway LR reaction, according to the manufacturer’s instructions. For overexpression of 14-3-3γ, mouse tyrosine 3-monooxygenase/tryptophan 5-monooxygenase activation protein gamma (YWHAG, GeneBank accession number NP_061359.2) was PCR-amplified from mouse brain cDNA using flanking primers containing BamHI and SalI restrictions sites and cloned into a pQE-30 vector to incorporate 6xHis-tag on its N-terminal. The protein sequence fused to 6xHis-tag was then subcloned with EcoRI and SalI restriction enzymes into the bicistronic vector pIRES2-GFP (BD Clontech), upstream of the internal ribosome entry site (IRES), allowing simultaneous expression of green fluorescence protein (GFP) and 14-3-3γ. To obtain pAd-CMV-6xHis-14G-IRES-GFP, full sequence containing CMV/6xHis14-3-3γ and IRES/GFP was subcloned into pENTR™ vector using EcoRI and NotI restriction enzymes and subsequently recombined into pAd/BLOCK-iT™-DEST, as described before.

Adenoviruses were produced by transfecting adenoviral vectors, linearized with PacI, into Human Embryonic Kidney 293 cell line, variant A (HEK293A) using Lipofectamine 2000 (Invitrogen). After 12 days of incubation, both cells and supernatant were harvested and subjected to three freeze-thaw cycles to facilitate adenovirus release. The suspension was clarified by centrifugation at 8000 *g* for 10 min at 4°C. The supernatant was then divided into 1-mL aliquots and stored at -80°C. A second round of amplification in HEK293A cells was performed to achieve an optimal viral load of the adenoviruses used in this study. The adenoviral titer was determined by flow cytometry using an antibody against the adenovirus hexon protein (ab24240, Abcam), as described by Bottley et al. [33], and was expressed in focus-forming units per milliliter (FFU/mL).

### Osteogenic lineage specific stainings

TNAP staining was performed using a chromogenic substrate solution containing 5-bromo-4-chloro-3-indolyl phosphate (BCIP, Calbiochem) and nitro blue tetrazolium (NBT, VWR Chemicals BDH). After fixation with 4% paraformaldehyde (PFA) for 5 min at RT, cells were washed twice with permeabilization buffer (Phosphate-buffered saline -PBS-, 0.05% Tween-20) for 2.5 min each. Cells were then incubated under agitation with a reaction buffer (100 mM Tris-HCl pH 8.3, 5 mM MgCl_2_, 0.015% BCIP, and 0.03% NBT) for 1 h at RT in the dark. The color reaction was stopped by thoroughly washing with Milli-Q water. Stained cells were mounted with Mowiol 4-88 and subsequently observed and photographed using an inverted phase-contrast microscope (I80i; Nikon, Japan). Following the staining procedure, to assess TNAP activity, cells were incubated with p-nitrophenylphosphate (p-NPP, Calbiochem), which is hydrolyzed by TNAP to produce p-nitrophenol (p-NP), a yellow product measurable at 405 nm. Briefly, cells were fixed and permeabilized following the same protocol used for BCIP/NBT staining, and then washed with a 50 mM bicarbonate buffer (pH 9.6) containing 1 mM MgCl_2_. Next, 1 mL of a reaction mixture composed of 50 mM bicarbonate buffer (pH 9.6), 1 mM MgCl_2_, and 10 mM p-NPP substrate was added, and cells were incubated with gentle shaking at 30°C in the dark. Kinetic measurements were performed by taking 100 μL samples at various intervals over a 15-min period, followed by the addition of 50 µL of 3 N NaOH to stop the reaction. The absorbance of p-NP was measured at 405 nm using a MultiSkan™ FC microplate photometer (Thermo Fisher Scientific).

For Alizarin Red S staining, cells were fixed with 4% PFA for 20 min at room temperature (RT) and washed three times with PBS. The cells were subsequently stained under agitation with 40 mM Alizarin Red S (pH 4.1-4.3) for 20 min in the dark at RT. After staining, Alizarin Red S was removed, and the cells were thoroughly washed with Milli-Q water. Quantification was performed by extracting the dye with 10% v/v acetic acid, following the protocol described by Gregory et al. [34].

To perform the Aniline Blue staining, cells were fixed with 4% PFA for 20 min at RT, followed by vigorous washing with PBS. The cells were then incubated with 2.5% w/v Aniline Blue solution, using a volume sufficient to cover the monolayer, for 5 min. After staining, cells were thoroughly washed at least three times with Milli-Q water to remove dye excess and mounted with Mowiol 4-88 for analysis by optical microscopy.

### 14-3-3γ knockdown and overexpression in hASCs

hASCs at passages 2-3 were transduced with 200 FFU/cell of adenoviruses carrying either 14-3-3γ overexpression or shRNA constructs, one day before induction with osteogenic differentiation medium. Adenoviral transduction was performed in complete DMEM containing 10% FBS for 2 h at 37°C, 5% CO₂, followed by fresh medium replacement. For control of infection, cells were transduced with adenovirus expressing GFP. WB was performed to assess down-regulation or constitutive expression of 14-3-3γ.

### SDS-PAGE and WB analysis

Cells were washed three times with PBS and lysed by adding and resuspending in a lysis buffer (60 mM Tris-HCl pH 7.5; 2% w/v SDS). The lysates were ultrasonicated for 5 min in an ice-cold water bath, boiled for 5 min, and centrifuged at 14,000 *g* for 10 min at 4°C. The resulting supernatants were separated by 12% w/v SDS-PAGE gel, with 50 µg of total protein loaded per well, and electro-transferred onto PVDF membrane. After blocking with 5% skimmed milk in PBS for 1 h at room temperature, membranes were incubated overnight under gentle agitation at 4°C with the following primary antibodies: anti-RUNX2 mAb (1:1000, sc-390351, Santa Cruz Biotechnology), anti-Histidine mAb (1:2500, Ab18184, Abcam) and anti-14-3-3γ polyAb (1:5000) [35]. An anti-β-Tubulin mAb (1:1000, T-4026, Sigma Aldrich) was used as loading control. After three washes with 0.01% Tween-20/PBS, the membranes were probed for 1 h at room temperature with horseradish peroxidase (HRP)-conjugated goat anti-rabbit IgG (1:5000, W4011, Promega) or horse anti-mouse IgG (1:2000, PI2000, Vector Laboratories) secondary antibodies. Blots were then washed three times with 0.01% Tween-20/PBS, followed by three washes with PBS, and visualized using ECL reagents (Thermo Fisher Scientific). The protein bands intensities were quantified with the software Image Studio™ Lite 5.2 (LI-COR Biosciences).

### RNA extraction and quantitative real-time PCR

Total RNA was extracted using Bio-Zol™ (RA02, Productos Bio-Lógicos), following the manufacturer’s protocol. After verifying RNA integrity, 1 μg of RNA per sample was reverse transcribed into cDNA using M-MLV Reverse Transcriptase (EA13, Productos Bio-Lógicos). Real-time PCR was performed on a QuantStudio 6-flex Real-time PCR System (ThermoFisher Scientific) using Maxima SYBR Green qPCR Master Mix (K0251, Thermo Fisher Scientific). The thermal cycling conditions were as follows: initial denaturation at 95°C for 10 min, followed by 40 cycles of 95°C for 15 s, 60°C for 30 s, and 72°C for 30 s, with a final extension at 72°C for 30 s. Expression of human Bone Gamma Carboxyglutamate Protein (BGLAP) and Secreted Phosphoprotein 1 (SPP1) genes were normalized to β-actin (ACTB), and data were analyzed using the ΔΔCt method [36]. The primers were synthesized by Macrogen and their sequences are listed in Table 1.

**Table 1:** Primer sequences used for Quantitative Real-Time PCR.

Genes/Proteins	Sequence	Tm (°C)
BGLAP / Osteocalcin	F:5’-CACCGAGACACCATGAGAGC-3’	63.4
	R:5’-CGGATTGAGCTCACACACCT-3’	62.2
SPP1 / Osteopontin	F:5’-CTCCTAGCCCCACAGAATGC-3’	63.5
	R:5’-CTGTGGGGACAACTGGAGTG-3’	64.6
ACTB / β-actin	F:5’-TGACGTGGACATCCGCAAAG-3’	62.8
	R:5’-CTGGAAGGTGGACAGCGAGG-3’	67.0

### Immunofluorescence staining of 14-3-3γ

To study the subcellular localization of 14-3-3γ during osteogenic differentiation, hASCs cultured on 12 mm coverslips, either untreated or induced with osteogenic differentiation medium (ODM) for 12 days, were fixed with 4% PFA for 20 min at RT. Fixed cells were then washed three times with PBS and permeabilized using 0.3% v/v Triton X-100/PBS for 10 min at RT. After additional PBS washes, the cells were blocked in 10% bovine serum albumin (BSA)/PBS for 1 h at RT. Excess serum was removed by washing three times with 0.1% v/v Triton X-100/PBS. The slides were then incubated overnight at 4° C with anti-14-3-3γ (same as used in WB) and anti-calnexin (610523, BD Biosciences), both diluted 1:500 in a solution of 0.1% v/v Triton X-100/PBS and 2% v/v BSA. The following day, cells were washed three times with 0.1% v/v Triton X-100/PBS and incubated for 1 h at RT with Goat anti-rabbit IgG Alexa Fluor 488 (A-11008, Thermo Fisher Scientific) and Donkey anti-mouse IgG Alexa 594 (A-21203, Thermo Fisher Scientific), both diluted 1:250 in the same Triton/BSA solution. After washing three times with PBS, the cells were mounted using Antifade Mounting Medium with DAPI (H-1200, VECTOR). Fluorescent images were captured using an Olympus FluoView 1000 confocal microscope and analyzed using the Fiji-ImageJ2 open-source software.

### Sample preparation for proteomic analysis

Four biological replicates of hASCs infected with adenovirus expressing GFP or 14-3-3γ shRNA on day 12 after osteogenic differentiation were lysed in lysis buffer (60 mM Tris-HCl pH 7.5; 2% w/v SDS). Samples (30 μg) were separated in 12% SDS-polyacrylamide gels, until the dye front migrated 1 cm into the separating gel. Gels were stained in darkness with 15 mL of colloidal Coomassie solution (0.12% w/v Coomassie Brilliant Blue G-250; 10% v/v H3PO4; 10% w/v (NH4)2SO4; 20% v/v methanol) for 5 min under gentle agitation. After staining, 1 cm² sections corresponding to whole proteome of each sample were excised with a clean razor blade into 1-mm cubes, transferred to individual Eppendorf tubes, and digested with trypsin following the protocol described by Link and LaBaer [37].

### Label-free liquid chromatography-tandem mass spectrometry (LC-MS/MS) analysis

Peptides were separated using a nanoHPLC system Ultimate3000 (Thermo Scientific) with an EASY-Spray ES903 CV18 column (50 cm × 50 μm ID, PepMap RSLC C18). The mobile phase flow rate was 400 nL/min using 0.1% formic acid in water (solvent A) and 0.1% formic acid in acetonitrile (solvent B). The gradient profile was set as follows: 4–30% solvent B for 114 min, 30–80% solvent B for 14 min and 80% solvent B for 2 min. 3 μL of each sample were injected. MS analysis was performed using a Q-Exactive HF mass spectrometer (Thermo Scientific). For ionization, 1,9 kV of liquid junction voltage and 250°C capillary temperature was used. The full scan method employed a m/z 375–1600 mass selection, an Orbitrap resolution of 120,000 (at m/z 200), a target automatic gain control (AGC) value of 3 x e6, and maximum injection times of 100 ms. After the survey scan, the 7 most intense precursor ions were selected for MS/MS fragmentation. Fragmentation was performed with a normalized collision energy of 27 eV and MS/MS scans were acquired with a dynamic first mass, AGC target was 5 x e5, resolution of 30,000 (at m/z 200), intensity threshold of 4.0 x e4, isolation window of 1.4 m/z units and maximum IT was 200 ms. Charge state screening was enabled to reject unassigned, singly charged, and equal or more than six protonated ions. A dynamic exclusion time of 15 s was used to discriminate against previously selected ions.

### MS data analysis

MS data were analyzed with MaxQuant (V: 2.2.0.00) using standardized workflows. Mass spectra *.raw files were searched against a database from Homo sapiens (reviewed and unreviewed proteins), entry UP000005640 from Uniprot. Precursor and fragment mass tolerance were set to 10 ppm and 0.02 Da, respectively, allowing 2 missed cleavages. Fixed modification: carbamidomethylation of cysteines. Variable modifications: protein N-terminal acetylation and methionine oxidation. Further data processing and statistical tests were performed in Perseus v1.6.15.0 software (MaxQuant). Data were filtered to remove proteins identified by site, reverse hits and contaminants. Label-free quantification (LFQ) intensity values were log2 transformed. Only proteins with LFQ values present in 75% of samples in at least one experimental group were used for downstream analysis. Missing values were imputed based on normal distribution using default settings (width 0.3, downshift 1.8). A two-sample Student’s *t*-test (S0 0, permutation-based false discovery rate -FDR- of 0.05, with 250 randomizations) was applied for statistical analysis.

### Gene ontology (GO) enrichment analysis

The enrichment analysis of differentially regulated proteins was performed using the clusterProfiler package (v. 4.12.6) in RStudio (v. 2024.09.0), applying GO Biological Processes and Kyoto Encyclopedia of Genes and Genomes (KEGG) pathways as ontology sources [38, 39]. Gene symbols were converted to *ENTREZ* IDs using the org.Hs.eg.db package (v. 3.19.1). For GO analysis, the enrichGO function was used to identify significantly enriched biological processes, with redundant GO terms simplified using the GOSemSim package (v. 2.30.2); simplify function, cutoff = 0.5, method = “Rel”. Significant GO terms were defined as those with an adjusted *p*-value < 0.05 and a gene count > 4. For KEGG pathway analysis, the enrichKEGG function was employed with a Benjamini-Hochberg FDR correction (adjusted *p*-value cutoff < 0.01) to control for multiple testing. Results from both analyses were visualized as dot plots, in which each term’s significance was color-coded by the adjusted *p*-value, and dot size was scaled by gene count.

## Results

### Characterization of hASCs

The hASCs isolations used in this study were characterized by flow cytometer analysis (Supplementary Data, Fig. S1A), verifying that > 95% of the cells expressed the positive markers CD90, CD73 and CD105, and < 2% of the cells expressed negative markers for hAD-MSCs, similar to our previous reports [22, 31]. Also, differentiation to the three mesodermal lineages -adipogenesis, chondrogenesis and osteogenesis- was verified (Supplementary Data, Fig. S1B), as recommended by the IFATS [30, 31].

### 14-3-3γ levels fluctuate in a stage-specific manner during osteogenic differentiation of hASCs

Previously, we observed that in hASCs, 14-3-3γ levels are reduced after 21 days of osteogenic differentiation [22]. To further investigate this, we examined 14-3-3γ levels at different time intervals corresponding to each stage of osteogenic commitment: proliferation (days 3-5), extracellular matrix (ECM) maturation (days 7-10), and mineralization (days 14-21) [40].

**Fig. 1:**
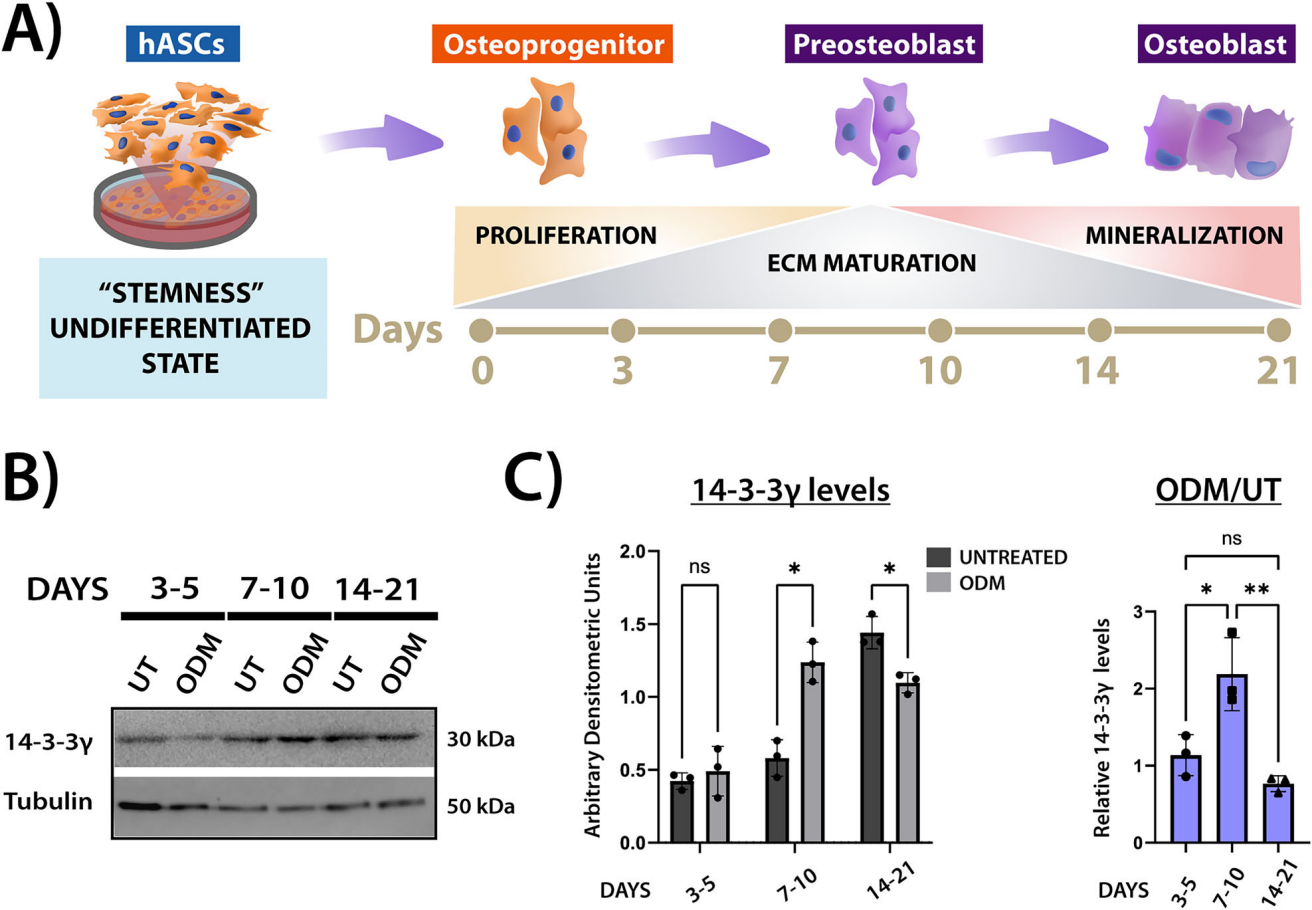
14-3-3γ relative levels during the three stages of osteogenic differentiation in hASCs. **A** Schematic representation and timeline of hASCs osteogenic differentiation, showing the transition from an undifferentiated state to osteoprogenitors, preosteoblasts, and mature osteoblasts across the three stages: proliferation (days 3–5), ECM maturation (days 7–10), and mineralization (days 14–21). **B** Samples (50 μg) of hASCs lysates from untreated (UT) and ODM-treated, at the three stages of osteogenic differentiation were separated by SDS/PAGE, and blots were divided for reaction with antibodies against 14-3-3γ or β-Tubulin (loading control). Data were similar in two other experiments. **C** Bar graphs showing densitometric quantification of 14-3-3γ levels normalized to β-tubulin. Left: Comparison between 14-3-3γ levels in UT and ODM-treated hASCs. Right: Ratio of 14-3-3γ levels in ODM-treated relative to UT hASCs at each time interval. Data represent mean ± SD from three independent experiments. Statistical significance was assessed by two-way ANOVA followed by Bonferroni *post hoc* test (left) and one-way ANOVA followed by Tukey’s *post hoc* test (right). *p < 0.05, **p < 0.01; ns: not significant.

WB analysis showed no significant differences in 14-3-3γ levels during the initial proliferation phase (Fig. 1B). However, during ECM maturation, 14-3-3γ levels increased up to two-folds in ODM induced-cells compared to untreated cells. Consistent with our previous observations, 14-3-3γ protein levels were lower in ODM-treated compared to untreated hASCs during the mineralization phase. These findings suggest that 14-3-3γ protein levels are regulated and finely tuned in a stage-specific manner, also suggesting specific roles for 14-3-3γ at each phase of osteogenesis.

### 14-3-3γ knockdown has stage-dependent effects on osteogenesis

**Fig. 2:**
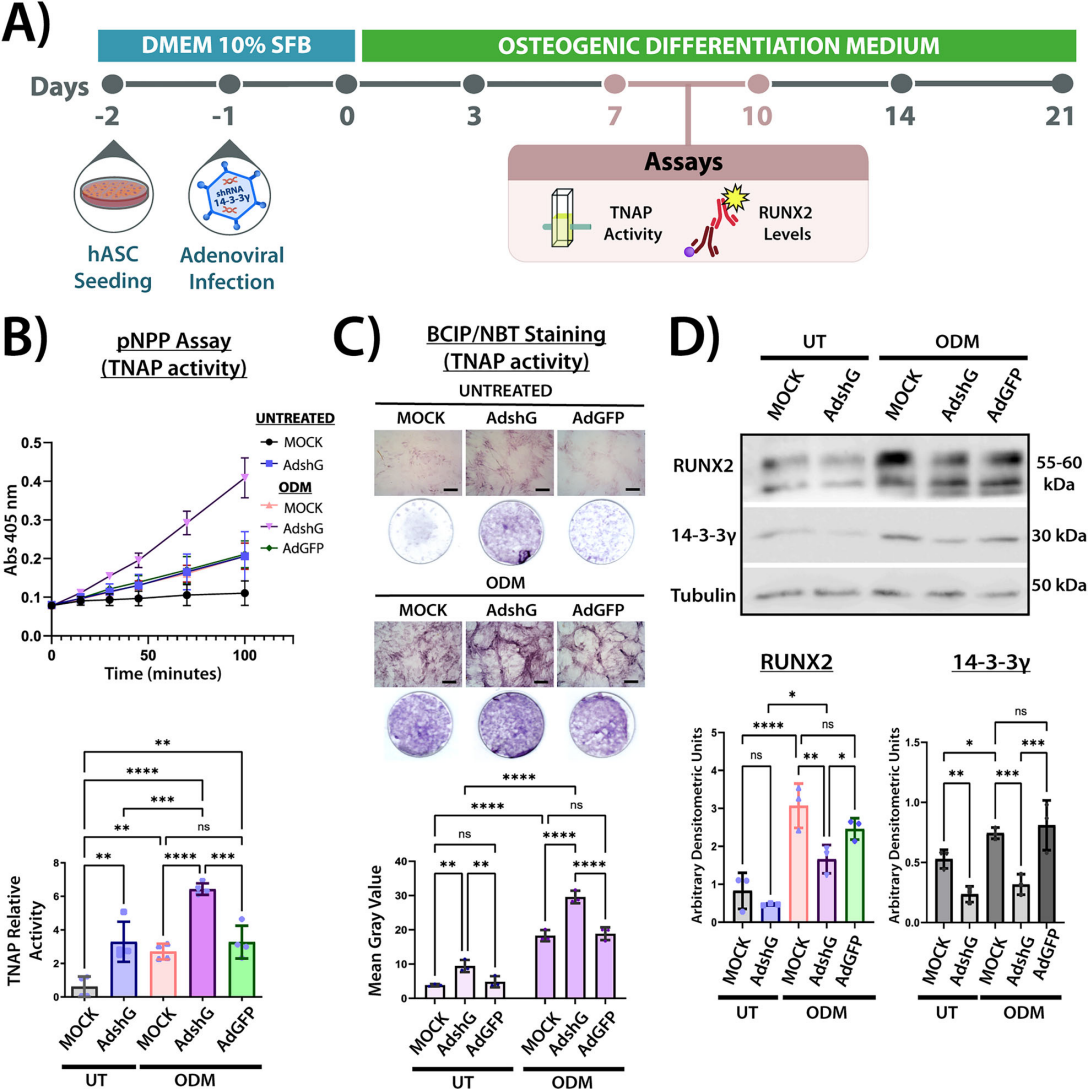
Effects of 14-3-3γ knockdown at the ECM maturation stage in osteogenic differentiation of hASCs. **Α** Schematic timeline of osteogenic differentiation in adenovirus-infected hASCs. TNAP activity, RUNX2 and 14-3-3γ protein levels were evaluated in a time interval between days 7–10. **B** TNAP activity assay using pNPP as substrate. Top: Absorbance at 405 nm over time in untreated: MOCK, Ad-shG-infected and ODM-treated: MOCK, Ad-shG-, and Ad-GFP infected hASCs. Bottom: Bars in the graph represent the TNAP relative activity calculated as the slope of each absorbance curve in the exponential phase of enzyme kinetics (shown on top). Data are presented as mean ± SD from four independent experiments (*n* = 4). Statistical differences were assessed by one-way ANOVA followed by Tukey’s *post hoc* test. **p < 0.01, ***p < 0.001, ****p < 0.0001. **C** Histochemical detection of TNAP activity by BCIP/NBT staining. Top: Representative bright-field images and macroscopic views of untreated: MOCK, Ad-shG-infected and ODM-treated: MOCK, Ad-shG-, and Ad-GFP-infected hASCs. TNAP activity is visualized as purple precipitates. Scale bars = 50 μm. Bottom: Bar graph represents mean gray values quantification from nine microscopy images per condition. Data are expressed as mean ± SD from three independent experiments (*n* = 3). Statistical analysis was performed using two-way ANOVA with Bonferroni’s *post hoc* test. **p < 0.01, ****p < 0.0001. **D** Samples (50 μg) of hASCs lysates from untreated: MOCK, Ad-shG-infected, and ODM-treated: MOCK, Ad-shG-, and Ad-GFP-infected were separated by SDS/PAGE, and blots were divided for reaction with specific antibodies against RUNX2, 14-3-3γ or β-Tubulin (loading control). Data were similar in two other experiments. Bar graphs show densitometric quantification of RUNX2 (Left) and 14-3-3γ (Right) normalized to β-tubulin. Data are shown as mean ± SD from three independent experiments (*n* = 3). Statistical significance was assessed by one-way ANOVA followed by Tukey’s *post hoc* test. *p < 0.05, **p < 0.01, ***p < 0.001, ****p < 0.0001; ns, not significant.

**Fig. 3:**
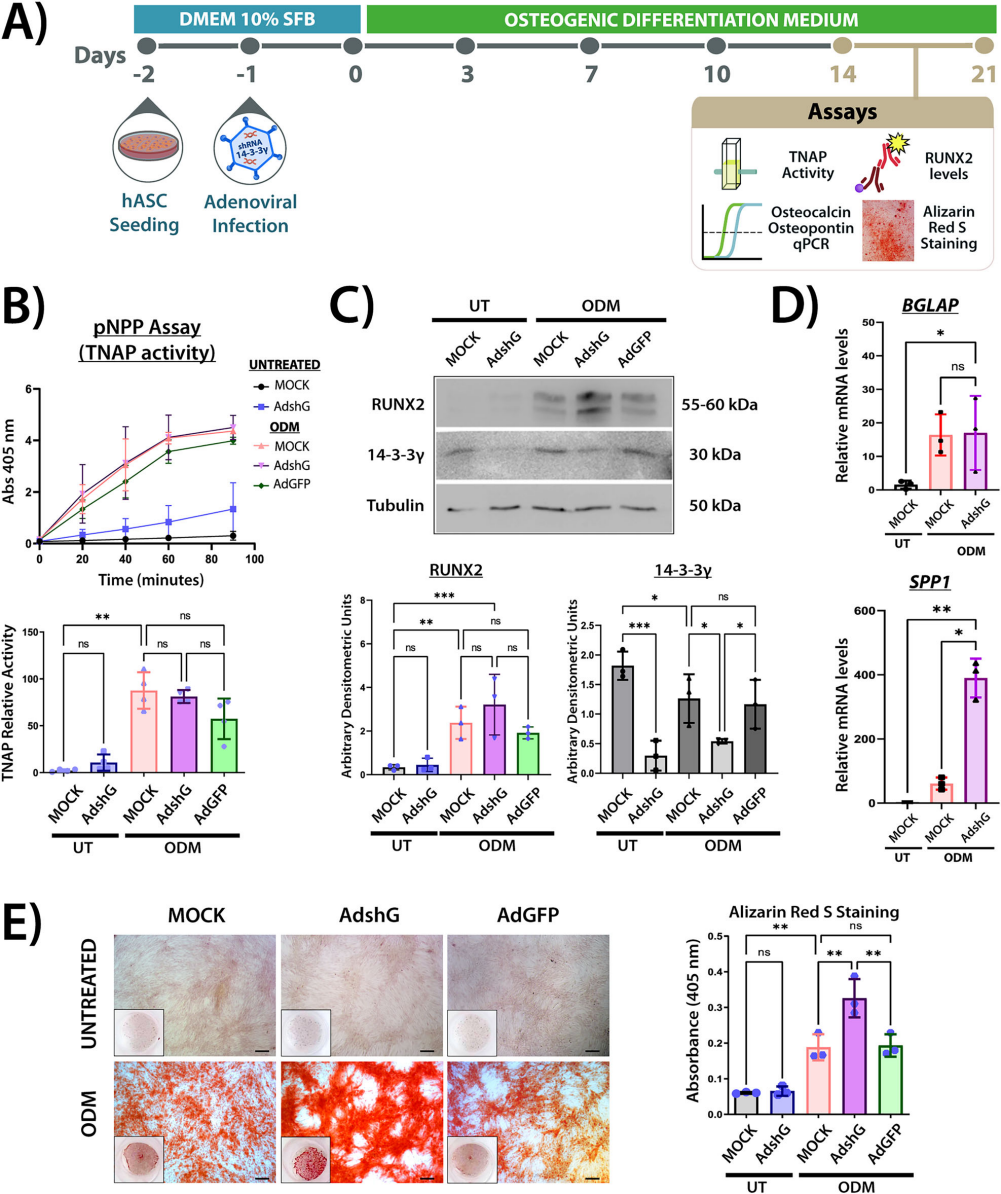
Effects of 14-3-3γ knockdown at the mineralization stage in osteogenic differentiation of hASCs. **A** Timeline of osteogenic differentiation in adenovirus-infected hASCs. The assays were performed between days 14-21. **B** TNAP activity assay (pNPP substrate). Top: Absorbance at 405 nm over time in untreated: MOCK, Ad-shG-infected and ODM-treated: MOCK, Ad-shG-, and Ad-GFP infected hASCs. Bottom: Bars graph represents the TNAP relative activity calculated as the slope of each absorbance curve in the exponential phase of enzyme kinetics (Top). Data are presented as mean ± SD from four independent experiments. Statistical analysis: one-way ANOVA followed by Tukey’s *post hoc* test. **C** Samples (50 μg) of hASCs lysates from the same conditions as in **B** were separated by SDS/PAGE, and blots were divided for reaction with specific antibodies against RUNX2, 14-3-3γ or β-Tubulin (loading control). Data were similar in two other experiments. Bar graphs, densitometry of RUNX2 (Left) and 14-3-3γ (Right) normalized to β-tubulin. Data are shown as mean ± SD from three independent experiments. Statistical analysis: one-way ANOVA followed by Tukey’s *post hoc* test. **D** Relative mRNA levels of osteocalcin (*BGLAP*) and osteopontin (*SPP1*) measured by qPCR at day 21 of osteogenesis, comparing MOCK and Ad-shG hASCs. Gene expression was normalized to the β-actin (*ACTB*). Bars represent mean data ± SD from three independent experiments. Statistical analysis: one-way ANOVA followed by Tukey’s *post hoc* test. **E** Calcium deposition analysis in the ECM by Alizarin Red S staining. Representative bright-field images and macroscopic views show red calcium-rich deposits in hASCs (as in B), at day 21. Scale bars = 50 μm. Bar graph shows the quantification of Alizarin Red S staining by absorbance measurement at 405 nm after dye extraction with acetic acid. Data are shown as mean ± SD from three independent experiments. Statistical analysis: one-way ANOVA with Tukey’s *post hoc* test. *p < 0.05, **p < 0.01, ***p < 0.001, ns, not significant.

To explore the functional role of 14-3-3γ during hASCs osteogenesis, we knockdown 14-3-3γ by using an adenovirus expressing shRNA against the 14-3-3γ paralog (AdshG), and compared the phenotype to the non-infected cells (MOCK) as well as cells infected with adenovirus expressing GFP (AdGFP) (Figs. 2A and 3A).

It was previously described that the upregulation of TNAP and RUNX2, two established early markers of the osteogenic program, indicate the beginning of the ECM maturation phase [6]. After 7–10 days of hASCs culture, TNAP activity was measured using tetrazolium salt. ODM treatment significantly increased TNAP activity compared to the untreated hASCs, with no significant differences observed in Ad-GFP-infected compared to MOCK cells (Fig. 2B). The 14-3-3γ knockdown increased TNAP activity, even in hASCs in the untreated condition, indicating that reduced 14-3-3γ levels enhanced TNAP activity during the maturation phase of osteogenic differentiation (Fig. 2B, C). In accordance with the TNAP activity results, RUNX2 levels were significantly higher in ODM-treated hASCs. However, in these cells, RUNX2 levels significantly decreased after 14-3-3γ knockdown (Ad-shG) compared to MOCK or Ad-GFP-infected cells (Fig. 2D). Notably, despite the downregulation of RUNX2 upon 14-3-3γ knockdown, in ODM-treated hASCs, RUNX2 levels remained higher than in untreated cells.

During the mineralization phase, RUNX2 protein levels and TNAP activity were significantly higher in ODM-treated cells between days 14 and 21, compared to untreated cells. However, no changes were observed as a result of 14-3-3γ knockdown during this phase (Fig. 3B, C).

To further explore the effects of reduced 14-3-3γ levels, we assessed the expression of two key non-collagenous extracellular matrix proteins, osteocalcin (encoded by *BGLAP* gene) and osteopontin (encoded by *SPP1* gene), which are upregulated by RUNX2 transcriptional activity during mineralization [41, 42]. Consistent with the RUNX2 protein levels data (Fig. 3C), the expression of both genes, *BGLAP* and *SPP1*, was significantly increased in MDO-treated compared to untreated hASCs. Interestingly, in Ad-shG cells, osteocalcin expression remained unchanged, whereas osteopontin expression showed a significant increase compared to MOCK (Fig. 3D).

Deposition of hydroxyapatite in the extracellular matrix was evidenced by Alizarin Red S staining [34]. As shown in Fig. 3E, calcium nodule formation was significantly higher in ODM-treated compared to untreated cells. Interestingly, 14-3-3γ knockdown significantly enhanced further calcium deposition, as it can be visualized by the more intense red staining (Fig. 3E, left), and also demonstrated by the extraction and quantification of the dye absorbance at 405 nm (Fig. 3E, right). These findings suggest that 14-3-3γ plays a critical role in modulating matrix mineralization, despite the lack of significant changes in other osteogenic markers at this stage.

### Label free LC-MS/MS analysis of Ad-shG and Ad-GFP hASCs

To investigate the underlying mechanisms responsible for the effects of 14-3-3γ knockdown during osteogenesis, we conducted an exploratory proteomic analysis at the onset of the mineralization phase (marked by the detection of early extracellular Ca²⁺ nodules using Alizarin Red S staining). To exclude changes attributable to adenoviral infection, we compared hASCs transduced with Ad-shG to those transduced with Ad-GFP. Proteins were extracted, as detailed before, after 12 days of ODM treatment and analyzed by mass spectrometry.

**Fig. 4:**
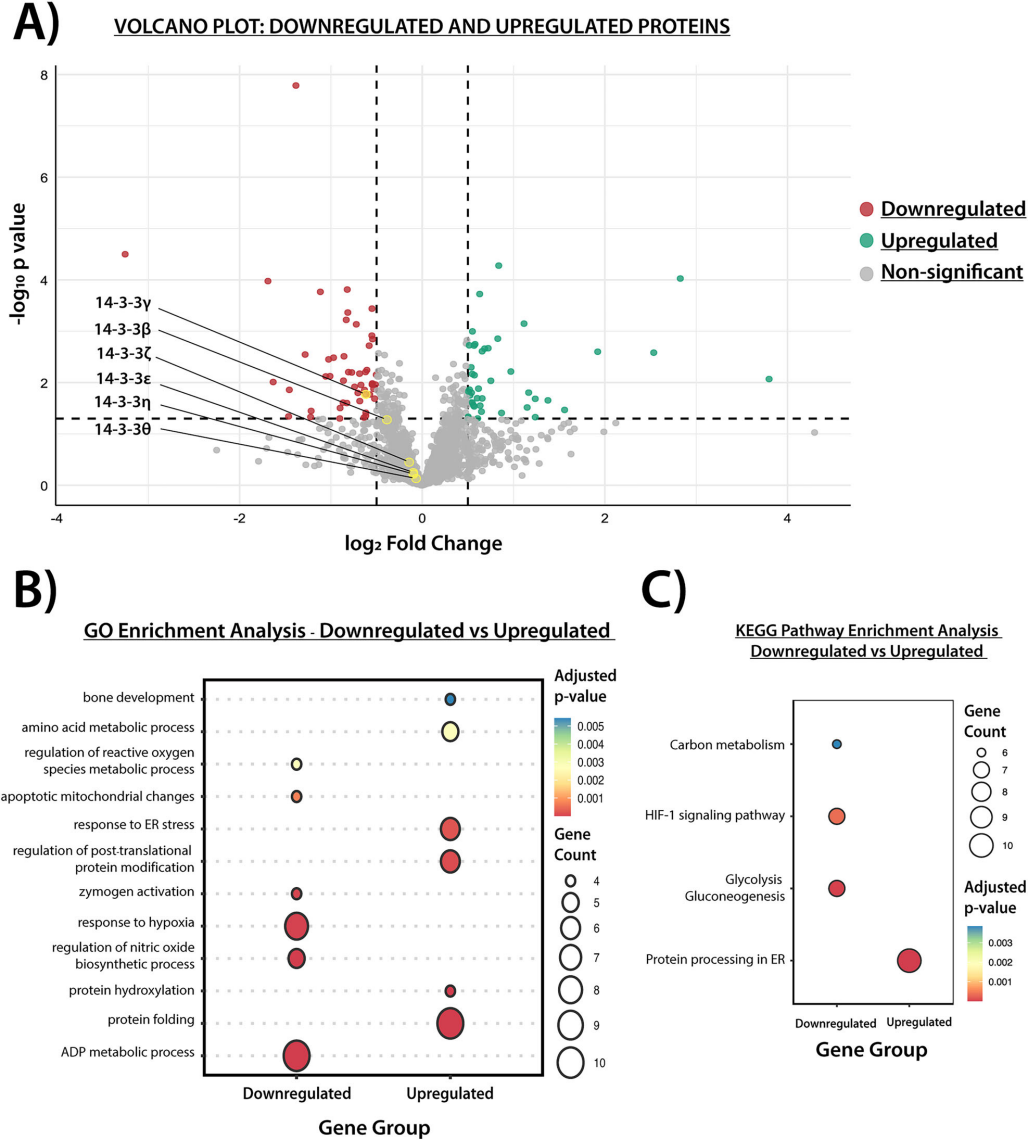
Proteomic analysis of 14-3-3γ knockdown hASCs at the onset of the osteogenic differentiation mineralization phase. **A** Volcano plot showing differentially regulated proteins in ODM-treated hASCs infected with Ad-shG relative to Ad-GFP-infected hASCs. Proteins are shown according to log₂ fold change (x-axis) and –log₁₀ *p*-value (y-axis). Dots represent upregulated (green), downregulated (red), and non-significantly (gray) regulated proteins. Yellow points indicate 14-3-3 paralogs, which are labeled. Statistical analysis was performed in Perseus (v1.6.15.0), and the plot was generated using the *ggplot2* package in R. **B** GO enrichment analysis. **C** KEGG pathway enrichment analysis of biological processes associated with upregulated and downregulated proteins. In **B** and **C**, the analysis was performed using the *clusterProfiler* package in R. Bubble size represents gene count per term, and color scale reflects adjusted *p*-values.

The samples exhibited normal distribution (Supplementary Data, Fig. S2A) and low variation of Pearson correlation values (ranging from 0.911 to 0.963, Supplementary Data, Fig. S2B), suggesting that 14-3-3γ knockdown did not significantly alter the overall proteome. However, the principal component analysis (PCA) showed that the replicates from each group (Ad-GFP or Ad-shG) clustered closely and were distinctly separated between them (Supplementary Data, Fig. S2C). We performed a Student’s *t* test with a permutation-based FDR of 0.05 to identify differentially regulated proteins, which were visualized by a Volcano plot (using as threshold a FC > 0.4 and *p* value < 0.05). From a total of 2147 identified proteins, 74 were downregulated (including 14-3-3γ itself) and 65 were upregulated in response to Ad-shG infection (Fig. 4A). Notably, no significant changes were observed in the relative abundances of other 14-3-3 paralogs, highlighting the specific role of 14-3-3γ in the previously described enhanced mineralization phenotype.

GO and KEGG pathway enrichment analyses were conducted using the clusterProfiler package in R to identify significantly enriched biological processes and molecular pathways associated with the upregulated and downregulated proteins following the knockdown of 14-3-3γ [38, 39]. The results indicated that downregulated proteins are predominantly associated with biological processes such as ADP metabolism, nitric oxide biosynthesis, and response to hypoxia (Fig. 4B). Consistently, enriched KEGG pathways for downregulated proteins included glycolysis/gluconeogenesis, the HIF-1 signaling pathway and carbon metabolism (Fig. 4C). In contrast, upregulated proteins are primarily linked to regulation of protein folding, hydroxylation, post-translational modifications, ER stress, amino acid metabolism, and bone development (Fig. 4B). This was further supported by KEGG pathway analysis, which showed enrichment for the protein processing in the ER (Fig. 4C).

To further understand the identified biological processes and interpret the spatial relationships between them, we analyzed the enriched subcellular localizations. Using the SubcellulaRVis tool [43], which relies on the Gene Ontology Cellular Component (GOCC) annotations, we identified both shared and specific localization patterns for down- and upregulated proteins in 14-3-3γ-knockdown hASCs (Supplementary Data, Fig. S3). The analysis revealed a common enrichment for both groups in the extracellular region, cytoplasm, and intracellular vesicles. In addition, downregulated proteins were specifically enriched in the lysosome (FDR: 5.8 ×10^-3^), vacuole (FDR: 1.3 ×10^-2^), and mitochondrion (FDR: 1.5 ×10^-2^), while upregulated proteins were particularly associated with processes in the ER (FDR: 1.38 ×10^-17^).

An in-depth literature review of the most significantly regulated proteins in 14-3-3γ-knockdown hASCs revealed that several have well-established roles in osteogenesis. This was summarized in the Supplementary Data (Table S1). Additionally, osteogenesis related proteins whose regulation did not reach statistical significance are included for reference (Table S2, Supplementary Data).

### Osteogenic differentiation induces 14-3-3γ subcellular localization enrichment in the ER

Given the overrepresentation of upregulated proteins localized to the ER following 14-3-3γ knockdown, we investigated whether the subcellular distribution of 14-3-3γ during osteogenic differentiation could provide insights into its role in this process.

**Fig. 5:**
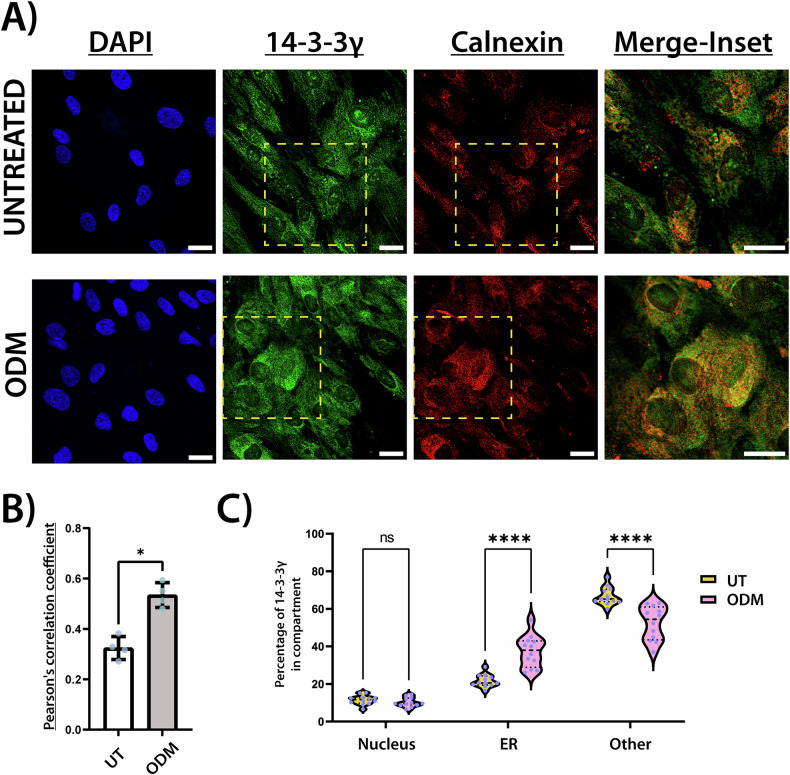
Subcellular distribution of 14-3-3γ at the onset of the osteogenic differentiation mineralization phase. **A** Confocal immunofluorescence microscopy images of untreated and ODM-treated hASCs at day 12. 14-3-3γ (green) and the ER marker calnexin (red) were detected by indirect immunofluorescence using anti-14-3-3γ (rabbit) and anti-calnexin (mouse) primary antibodies, followed by Alexa Fluor™ 488-anti-rabbit and Alexa Fluor™ 594-anti-mouse secondary antibodies, respectively. Nuclei were stained with DAPI (blue). Scale bars: 25 μm. Images are representative from four independent experiments. **B** Quantification of 14-3-3γ and calnexin colocalization using Pearson’s correlation coefficient, calculated with the JACoP plugin in FIJI software. Data are shown as mean ± SD from four independent experiments with three images per condition. Statistical significance was determined using a paired Student’s *t* test. *p < 0.05. **C** Percentage of 14-3-3γ fluorescence signal localized in the nucleus, ER, or other subcellular compartments in untreated and ODM-treated hASCs. Quantification was carried out in ImageJ using three 40× magnification images per condition from four independent experiments (*n* = 4). Statistical analysis was performed by two-way ANOVA followed by Bonferroni’s *post hoc* test. ****p < 0.0001; ns, not significant.

In untreated hASCs, 14-3-3γ is distributed ubiquitously throughout the cell (Fig. 5). However, upon osteogenic induction, 14-3-3γ showed a peri-ER distribution, as evidenced by increased colocalization with calnexin, a specific ER marker (Fig. 5).

### 14-3-3γ Overexpression suppresses matrix mineralization and collagen deposition

**Fig. 6:**
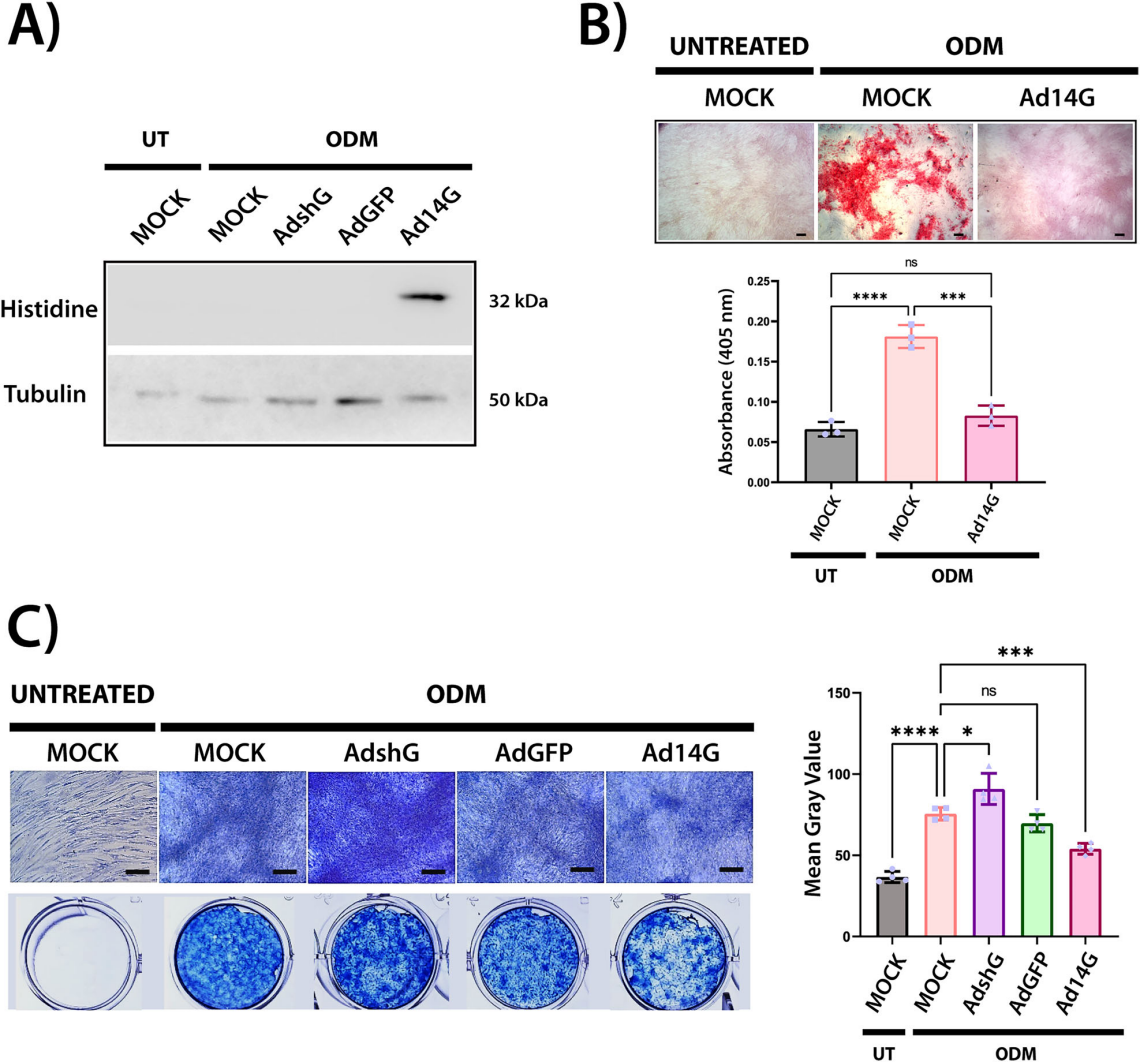
Effects of 14-3-3γ overexpression on matrix mineralization and collagen deposition in osteogenic differentiation of hASCs. **A** Western blot showing recombinant 14-3-3γ protein expression at day 21 of osteogenic differentiation of hASCs. Samples (50 μg) of hASCs lysates from untreated: MOCK, and ODM-treated: MOCK, Ad-shG-, Ad-GFP-, and Ad-6xHis-14G-IRES-GFP-infected hASCs were separated by SDS/PAGE, and blots were divided for reaction with specific antibodies against Histidine-tag (to detect recombinant 14-3-3γ) or β-Tubulin (loading control). Data were similar in two other experiments. **B** Calcium deposition analysis by Alizarin Red S staining at 21 days of osteogenic differentiation. Top: Representative bright-field images of untreated (UT) and ODM-treated: MOCK and Ad-6xHis-14G-IRES-GFP-infected hASCs. Scale bars: 50 μm. Bottom: Quantification of Alizarin Red S staining by absorbance at 405 nm after dye extraction with acetic acid. Data represent mean ± SD from three independent experiments (*n* = 3). Statistical significance was assessed using one-way ANOVA followed by Tukey’s *post hoc* test. ***p < 0.001, ****p < 0.0001; ns, not significant. **C** Collagen deposition analysis by Aniline Blue staining at 12 days of hASCs osteogenic differentiation. Left-top: Representative bright-field images of untreated: MOCK, and ODM-treated: MOCK, Ad-shG-, Ad-GFP-, or Ad-6xHis-14G-IRES-GFP infected hASCs Scale bars: 50 μm. Left-bottom: macroscopic views of the corresponding stained wells. Right: Bar graph shows quantification of mean gray intensity from four independent experiments. Statistical significance was assessed using one-way ANOVA with Tukey’s *post hoc* test. *p < 0.05, ***p < 0.001, ***p < 0.0001; ns not significant.

To further investigate the role of 14-3-3γ during osteogenesis of hASCs, we used an adenoviral vector (pAd-CMV-6xHis-14G-IRES-GFP) for its constitutive overexpression in hASCs, and examined its effects on the mineralization phase. The expression of the recombinant 14-3-3γ was confirmed by WB analysis (Fig. 6A).

After 14 days of osteogenic differentiation, calcium deposition was quantified by Alizarin Red S staining. In contrast to our previous results for 14-3-3 knockdown, hASCs overexpressing recombinant 14-3-3γ showed a significant decrease in calcium deposition, in comparison to MOCK ODM-treated cells (Fig. 6B).

To determine whether the effect of modulating 14-3-3γ levels impacts late osteogenesis beyond mineral deposition, we determined collagen accumulation by Aniline Blue staining (Fig. 6C). Consistent with the Alizarin Red S staining findings, 14-3-3γ knockdown enhanced collagen accumulation, as evidenced by more intense Aniline Blue staining, whereas its overexpression resulted in a significant decrease in collagen matrix deposition.

## Discussion

Osteogenesis is a dynamic process characterized by a tightly coordinated sequence of molecular and biochemical events [3–5]. Despite advances in the field, our understanding of how these mechanisms are regulated at each stage of osteoblast development and how multiple signaling pathways crosstalk is limited. Given their ability to modulate a broad range of client proteins, 14-3-3 proteins have emerged as potential points of convergence of these regulatory networks [13]. This interest has gained relevance as increasing evidence links aberrant expression of 14-3-3 paralogs to the pathology of various bone disorders [12]. In this context, proper regulation of 14-3-3 protein abundance appears to be essential for maintaining skeletal homeostasis. Our previous work supported this notion by showing that 14-3-3β, γ and ε are differentially regulated during the late stage of osteogenic differentiation of hASCs, suggesting paralog-specific contributions to this process [22]. Here, we focused on 14-3-3γ and investigated its protein levels dynamics throughout the progression of osteogenesis. WB analysis revealed a transient increase in 14-3-3γ levels at the onset of ECM maturation (Fig. 1C), followed by a significant downregulation during the mineralization phase. This biphasic regulation profile suggests that 14-3-3γ may act as a modulator of key molecular events during both early and late stages of osteogenesis. To further explore this possibility, we assessed the effect of 14-3-3γ knockdown on cellular processes associated with ECM maturation and matrix mineralization.

We analyzed two well-established markers of early osteogenic commitment, RUNX2 and TNAP [6]. Intriguingly, while 14-3-3γ knockdown in ODM-treated hASCs led to an increase in TNAP activity, RUNX2 protein levels were significantly decreased (Fig. 2B–D), although higher than in untreated cells. This discrepancy between the effects on RUNX2 and TNAP is striking, but not unprecedented: other upstream regulators capable of modulating TNAP expression independently of RUNX2 have been described [44–46].

As differentiation progressed into the mineralization phase, both TNAP activity and RUNX2 protein levels remained elevated in response to ODM (Fig. 3B and C), with a greater magnitude compared to the earlier stage. These findings are consistent with previous reports in hASCs [47, 48]. Notably, 14-3-3γ knockdown did not produce significant changes in TNAP or RUNX2 at this stage. Given their reciprocal regulation, the normalization of RUNX2 levels may result from the increased TNAP activity observed during the preceding ECM maturation phase after 14-3-3 knockdown (Fig. 2B, C). This suggests that the influence of 14-3-3γ on these markers depends on the stage of the osteogenic differentiation. We next assessed the mRNA expression of the two most abundant non-collagenous proteins, osteocalcin and osteopontin, by qPCR [49]. Interestingly, osteopontin expression was significantly increased by 14-3-3γ knockdown after 21 days of osteogenic induction (Fig. 3D), whereas osteocalcin levels remained unaltered. Despite the absence of changes in RUNX2 levels, Alizarin Red S staining showed a significant increase in calcium deposition in 14-3-3γ knockdown hASCs under osteogenic conditions (Fig. 3E).

To investigate the molecular mechanisms underlying the phenotypes observed upon 14-3-3γ knockdown during osteogenesis of hASCs, we conducted an exploratory proteomic analysis at the onset of the mineralization phase. Functional enrichment analysis revealed that 14-3-3γ knockdown led to changes in biological processes associated with energy metabolism and the regulation of reactive oxygen species. Furthermore, proteins involved in the ER stress response were also enriched (Fig. 4B, C). This finding is particularly relevant given the critical role of the ER during osteogenesis, where it sustains the high levels of protein synthesis, folding, and secretion, while also contributing to the calcium-phosphate cluster formation required for mineralization [50–52]. Upon osteogenic induction, 14-3-3γ redistribution to the ER (Fig. 5) suggests its potential role in ER-associated processes during osteogenesis. Such roles may include the regulation of protein trafficking, assistance in quality control, or ER retention through the masking of targeting motifs on client proteins [53]. It is also possible that 14-3-3γ redistribution could be associated with the mitochondria, given the metabolic changes observed here (Fig. 4B), and the known functional association between the ER and mitochondria during the osteogenic differentiation [52].

The enhanced mineralization phenotype observed upon 14-3-3γ knockdown could be explained by the differential regulation of proteins —both upregulated and downregulated— with known roles in osteogenesis (Supplementary Data, Table S1). Some of these changes mirror the normal expression trends of these proteins during terminal osteogenic differentiation, supporting the notion that 14-3-3γ knockdown accelerates this process. Although no significant changes were detected in the core components of type I collagen (COL1A1/COL1A2) (Supplementary Data, Table S2), we identified a significant upregulation of a network of proteins involved in type I collagen proteostasis, including its folding, quality control, secretion, and extracellular assembly (Supplementary Data, Table S1). Among these proteins, osteonectin stood out for its multifaceted role in bone formation [54, 55]. Through its collagen- and calcium-binding domains, osteonectin contributes to ECM organization by regulating collagen assembly and promoting hydroxyapatite formation. Although the interaction of osteonectin with 14-3-3γ remains to be investigated, a previous study in fibroblasts showed that 14-3-3σ interacts with osteonectin and modulates its activity in the ECM, ultimately affecting negatively collagen type I deposition [56]. Considering that 14-3-3σ is primarily expressed in epithelial tissues, it is possible that 14-3-3γ may exert a similar regulatory function in osteoblasts.

To assess whether the increased abundance of proteins associated with collagen type I proteostasis and ER stress in 14-3-3γ knockdown hASCs are linked with impaired collagen secretion into the ECM, we performed Aniline Blue staining. Notably, 14-3-3γ knockdown resulted in more intense Aniline Blue staining (Fig. 6C), suggesting that the upregulation of collagen-processing machinery contributes to an enhanced deposition of collagen into the ECM.

Finally, to evaluate whether sustained overexpression of 14-3-3γ affects mineralization, we analyzed calcium deposition by Alizarin Red S staining and collagen deposition by Aniline Blue. As expected, maintaining high levels of 14-3-3γ throughout the differentiation period impaired both processes (Fig. 6B, C), further supporting a critical role for the regulated levels of 14-3-3γ in osteogenic progression. These results are consistent with previous findings by Sun et al., who reported a correlation between increased 14-3-3γ expression and reduced osteogenic potential in extensively passaged MSCs [26].

Kim et al. [57] demonstrated that 14-3-3γ is essential for postnatal development and survival in mice, as none of the 242 pups born with homozygous knockout of the gene survived past weaning. Furthermore, heterozygous 14-3-3γ knockout mice exhibited significantly reduced body size and weight compared to their wild-type littermates, highlighting a dosage-sensitive role for this paralog. In humans, haploinsufficiency of YWHAG —the gene encoding the 14-3-3γ paralog— has been implicated in a spectrum of neurodevelopmental disorders, including intellectual disability, early-onset epilepsy, global developmental delay, hypotonia, and variable behavioral phenotypes [58–60]. These phenotypes are thought to result from disruption of 14-3-3γ–mediated signaling pathways, although the precise molecular mechanisms remain largely unresolved. Notably, structural analyses of pathogenic YWHAG missense variants have revealed clustering within the phosphopeptide-binding groove (10 of 12 known pathogenic mutations) and at residues critical for dimerization, indicating a molecular effect [59].

There is evidence linking hypermineralization (observed here upon 14-3-3γ knockdown) to developmental delays, particularly in certain genetic syndromes. Although hypomineralization is more commonly associated with developmental delay, several disorders demonstrate that hypermineralization can also impair normal growth and neurodevelopment. For example, infantile malignant osteopetrosis is characterized by excessive bone density due to defective osteoclast function, leading to cranial nerve compression, vision and hearing loss, and global developmental delay [61, 62]. Similarly, sclerosing bone dysplasias such as LRP5-related osteosclerosis-developmental delay syndrome present with increased bone mineralization, craniosynostosis, and cognitive impairment [63]. Another example is Pycnodysostosis, caused by mutations in CTSK, where dense but brittle bones are accompanied by short stature, delayed closure of cranial sutures, and psychomotor delay [64]. These examples illustrate potential links between our findings and the observed phenotypes suffered by mice and humans with YWHAG haploinsufficiency. Although our results, taken together with previous literature, strongly suggest that the mineralization increased after 14-3-3γ KD is in the context of osteogenic differentiation, we cannot completely exclude the possibility that pathologic calcification may be involved.

## Conclusions

Our findings demonstrate that the 14-3-3γ paralog functions as a negative regulator of extracellular matrix mineralization during the osteogenic differentiation of hASCs. Its knockdown significantly enhances TNAP activity and promotes both calcium and collagen deposition, indicating that 14-3-3γ constraints osteoblast maturation. Moreover, the subcellular redistribution of 14-3-3γ toward the ER upon osteogenic induction, together with proteomic evidence linking its downregulation to bone development and ER stress-related pathways, suggests a specific role in coordinating cellular homeostasis during osteogenic differentiation. Given the druggability of 14-3-3 proteins [15, 65–67], this information holds significant promise for the discovery of novel therapeutic strategies for numerous genetic and age-associated bone remodeling related disorders.

## Supplementary information

Supplementary Data of 14-3-3g Knockdown Promotes Matrix Mineralization in human Mesenchymal Stromal Cells

Related Manuscript File

## Data Availability

The datasets used and/or analyzed during the current study are available from the corresponding author on reasonable request.
