# Supplementary material for: 14-3-3γ Knockdown promotes matrix mineralization in human mesenchymal stromal cells
Source: Cell Death Dis. 2026 Mar 27;17(1):415. doi: 10.1038/s41419-026-08540-4 (PMC13149861; doi:10.1038/s41419-026-08540-4)
Supplement: Supplementary file 1 — Supplementary Data of 14-3-3g Knockdown Promotes Matrix Mineralization in human Mesenchymal Stromal Cells [file 41419_2026_8540_MOESM1_ESM.docx]

**Supplementary Data - Supplementary Information**

**14-3-3γ Knockdown Promotes Matrix Mineralization in human Mesenchymal Stromal Cells**

Lautaro Rivera^1^, Sergio Müller^1^, Marina Uhart^*1^, and Diego Martin Bustos^*1^.

* Corresponding authors with equal contribution.

1. Instituto de Histología y Embriología de Mendoza (IHEM, Universidad Nacional de Cuyo, CONICET), Mendoza, Argentina

2. Facultad de Ciencias Exactas y Naturales, Universidad Nacional de Cuyo, Mendoza, Argentina.

**This file includes:**

Supplementary figures S1 to S3

Supplementary table 1 (Table S1)

Supplementary table 2 (Table S2)

Supplementary figure S4

**Supplementary figures**

**Figure S1**


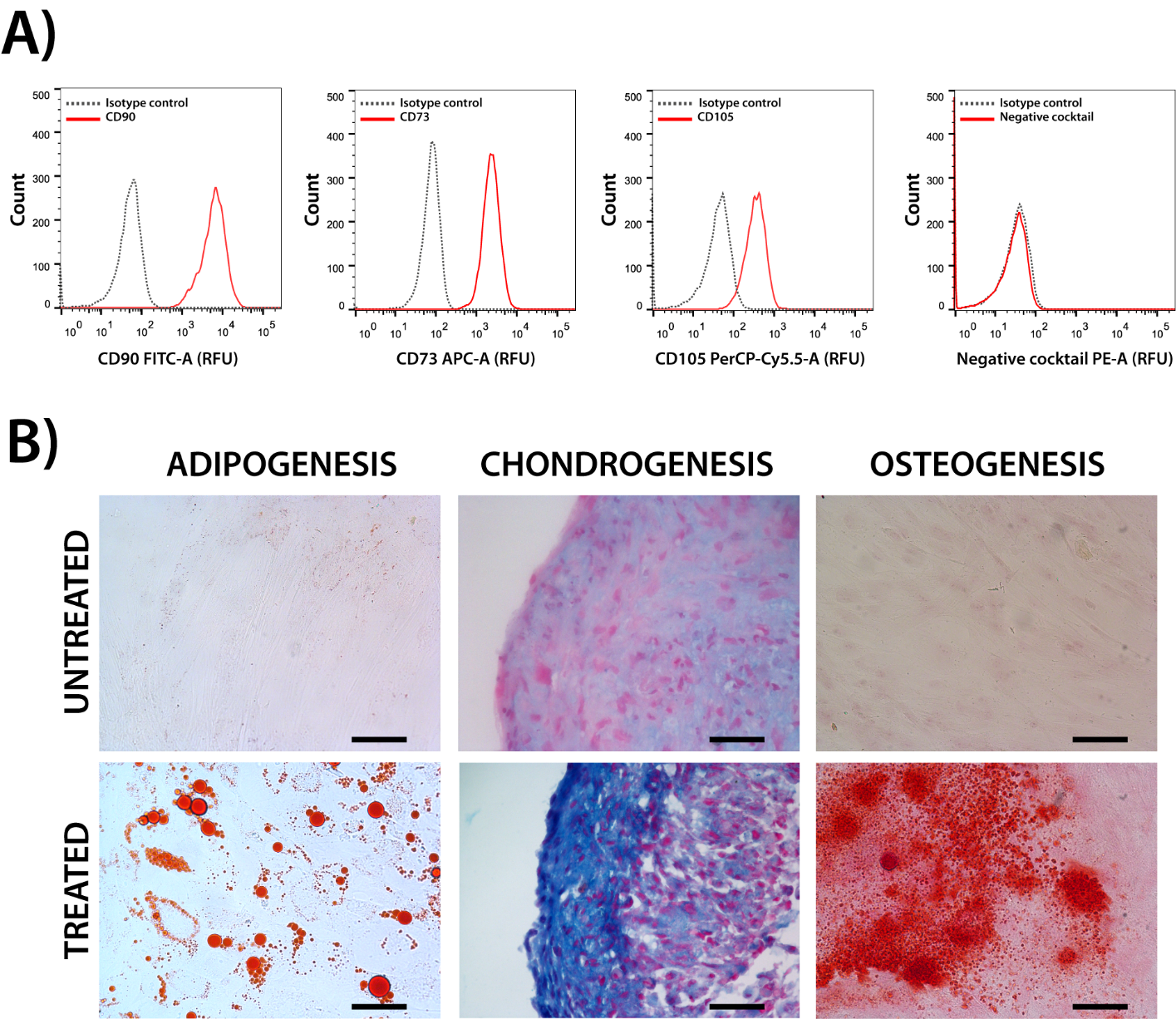


**Figure S1. Characterization of human adipose-derived mesenchymal stromal cells (hASCs).** A) Representative flow cytometry histograms showing the expression of positive MSC surface markers (CD73, CD90, CD105) and negative markers (negative cocktail) in Relative Fluorescence Units. Red solid lines represent antibody-stained samples; black dashed lines indicate isotype controls for each fluorochrome-conjugated antibody. B) Representative bright-field images of hASC differentiation toward adipogenic, chondrogenic, and osteogenic lineages. Left: Oil Red O staining of intracellular lipid droplets at day 10 of adipogenic differentiation. Middle: Alcian Blue staining of extracellular proteoglycans at day 21 of chondrogenic differentiation. Nuclei were stained with Nuclear Fast Red (red). Right: Alizarin Red staining of calcium phosphate deposits at day 21 of osteogenic differentiation. Scale bars: 50 μm. Images shown are representative of a single donor**.**

**Figure S2**

**
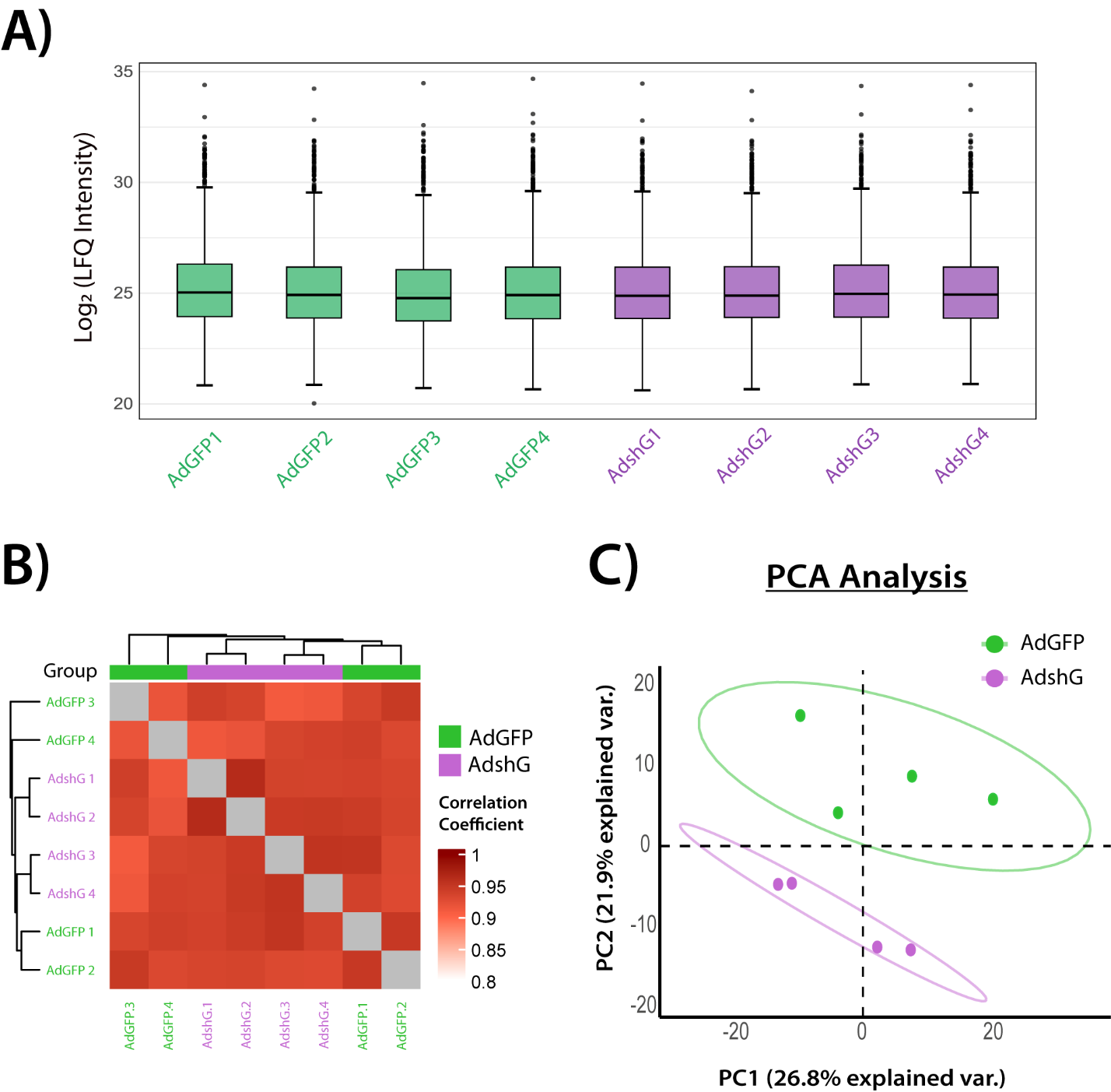
**

**Figure S2. Proteomic data quality analysis following 14-3-3γ knockdown in hASCs.** A) Box-plot showing the distribution of log₂-transformed label-free quantification (LFQ) intensities for the 2147 proteins identified across all samples. The analysis was performed using the Perseus software v1.6.15.0 (MaxQuant). B) Hierarchical clustering heatmap based on Pearson correlation coefficients between all samples from Ad-GFP– and Ad-shG–infected hASCs. The color scale ranges from a minimum correlation of 0.8 to a maximum of 1. The analysis was performed using the Perseus software v1.6.15.0 (MaxQuant). C) Principal component analysis (PCA) showing variance between Ad-shG (purple dots) and Ad-GFP (green dots) sample groups. PCA was performed in Perseus software v1.6.15.0 (MaxQuant).

**Figure S3**

**
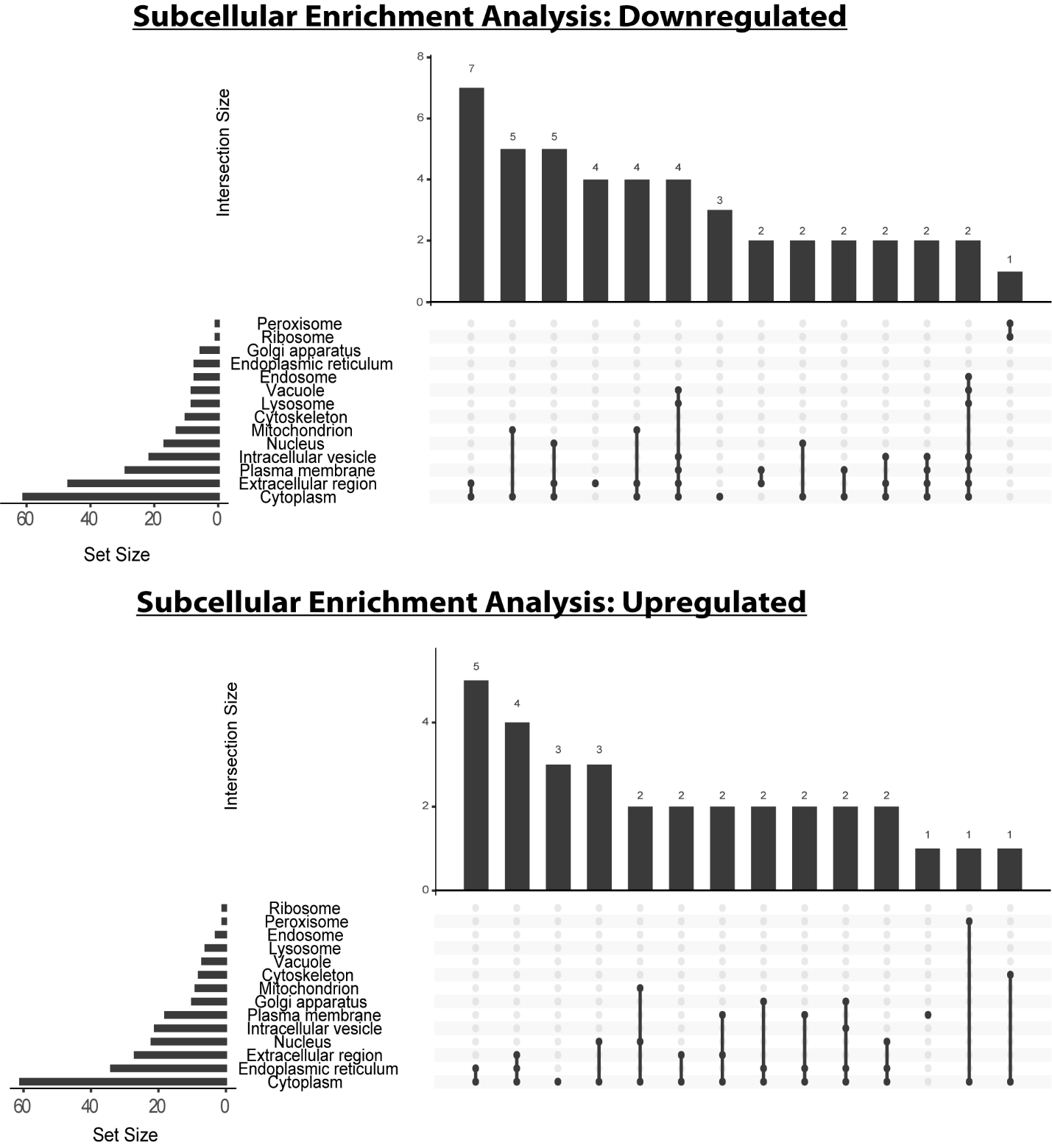
**

**Figure S3. Subcellular enrichment analysis following 14-3-3γ knockdown in hASCs.** Subcellular compartment enrichment analysis of downregulated (top) and upregulated (bottom) proteins in Ad-shG *versus* Ad-GFP samples. Barplots indicate intersection sizes across annotated subcellular localizations, highlighting organelles enriched among differentially regulated proteins. The analysis was performed using the SubcellulaRVis web tool, which integrates Gene Ontology Cellular Component (GOCC) annotations.

**Table S1**

| GENE | PROTEIN | MOLECULAR FUNCTION | Log_2_  Fold  Change | REFERENCE |
| --- | --- | --- | --- | --- |
| *AKR1C1* | Aldo-Keto Reductase Family 1 Member C1 | *Protein involved in the metabolism of aldehydes and ketones. It plays an essential role in the metabolism of steroid hormones such as progesterone and androsterone.*  ○ It is upregulated during the early stages of osteogenesis, with expression levels decreasing as differentiation progresses.  ○ AKR1C1 acts as a negative regulator of osteogenesis in hASCs through modulation of the progesterone receptor.  ○ AKR1C1 knockdown enhances osteogenic differentiation, as evidenced by increased TNAP activity, elevated RUNX2 levels, and greater mineralization. | **-0,82** | (1) |
| *CAV1* | Caveolin 1 | *Scaffolding protein of cholesterol-rich caveolae lipid rafts in the plasma membrane*  *○* CAV1 knockdown enhances TNAP activity and mineralization (as assessed by Alizarin Red S staining) in bone marrow-derived mesenchymal stem cells  ○ Caveolin-1 expression increases progressively during osteogenesis.  ○ Caveolin-1 knockout mice display increased bone volume during postnatal growth. | **-0,42** | (2–4) |
| *CNN1* | Calponin 1 | *Protein associated to actin filaments*  ○ Calponin-1 knockout mice exhibit increased bone formation.  ○ Calponin-1 overexpression inhibits late-stage osteogenic differentiation and mineralization of osteoblasts in *in vitro* cultures. | **-1,11** | (5,6) |
| *PTX3* | Pentraxin 3 | *Secreted glycoprotein induced in response to inflammation*  ○ Primary mouse osteoblasts express low levels of PTX3 under non-inflammatory conditions, and its absence does not affect collagen deposition or hydroxyapatite crystal formation.  ○ Under inflammatory conditions, Pentraxin 3 promotes osteogenesis in both primary osteoblasts and MC3T3-E1 pre-osteoblasts.  ○ It is upregulated during early osteogenesis (day 3), as shown by secretome analysis of hASCs. | **-0,58** | (7) |
| *MAPK3* | ERK1 | *Member of the MAP kinase family*  ○ Osteogenic differentiation of MSCs involves ERK activation during the early stage, followed by inactivation during intermediate and late stages. Sustained ERK activation, induced by TRIB3 knockdown, inhibits osteogenic differentiation.  ○ ERK inhibitors assays indicate that the MAPK pathway plays a positive regulatory role during early osteogenesis but acts as a negative regulator during the later stages of differentiation. | **-0,26** | (8–10) |
| *SLC2A1* | GLUT1 | *Major glucose transporter in bone*  ○ Relative GLUT1 levels decrease during osteogenesis of mesenchymal stem cells derived from bone marrow and dental pulp.  ○ GLUT1 is essential for the induction of type I collagen synthesis, preceding the upregulation of RUNX2.  ○ GLUT1 inhibits RUNX2 ubiquitination.  ○ GLUT1 inhibition by phloretin enhances the expression of osteogenic genes. | **-0,68** | (11–13) |
| *SORBS3* | Vinexin | *Plasma membrane-associated protein involved in the formation of focal adhesions*  ○ Vinexin depletion increases TNAP expression and enhances calcification, as evidenced by Alizarin Red S staining in MSCs. | **-0,25** | (14) |
| *SOD2* | Superoxide Dismutase 2 | *Catalyzes the dismutation of superoxide radicals into hydrogen peroxide and oxygen within mitochondria.*  ○ SOD2 expression is upregulated during osteogenesis to reduce excessive production of reactive oxygen species (ROS).  ○ SOD2 knockdown leads to mitochondrial ROS accumulation and impairs osteogenesis, as indicated by TNAP and Alizarin Red S staining. | **-0,8** | (15–18) |
| *LOX* | Lysyl Oxidase | *Catalyzes the cross-linking of collagen and elastin.*  *○* Lysyl oxidase knockdown enhances BMP4-induced osteogenesis through activation of the *Wnt* signaling pathway.  ○ LOX inhibition promotes bone formation *in vivo* | **-0,25** | (19) |
| *SPARC* | Osteonectin | *Calcium-binding glycoprotein secreted into the extracellular matrix during bone formation*  ○ Osteonectin binds strongly to hydroxyapatite crystals and type I collagen.  ○ Osteonectin knockout results in reduced accumulation of mature type I collagen. Elevated osteonectin levels are associated with enhanced collagen deposition in the extracellular matrix.  ○ SPARC knockout impairs osteogenesis in boneMSCs. | **+0,61** | (20–23) |
| *TGM2* | Transglutaminase 2 | *Secreted enzyme involved in the cross-linking and stabilization of ECM proteins.*  ○ TGM2 knockdown impairs the expression of TNAP, osteocalcin, and RUNX2 in bone marrow derived MSCs, whereas its overexpression has the opposite effect.  ○ Treatment with forskolin promotes osteogenesis by inducing a conformational change in TGM2, enhancing its activity on target proteins.  ○ Inhibition of its activity disrupts type I collagen accumulation in the ECM and impairs mineralization. | **+0,52** | (24–26) |
| *HTRA1* | HtrA1 | Serine protease  ○ Produced and secreted by both osteoblasts and osteoclasts.  ○ Specific proteolytic substrates include decorin and biglycan (but not type I collagen), highlighting its critical role in ECM homeostasis.  ○ HTRA1 expression increases during osteogenic induction of both human and mouse mesenchymal stem cells.  ○ HTRA1 silencing impairs mineralization and TNAP activity in bone marrow derived MSCs, without affecting RUNX2 or osteopontin expression, while promoting adipogenesis.  ○ Recombinant HTRA1 protein enhances mineralization.  ○ HTRA1 is a direct transcriptional target of RUNX2.  ○ Lentiviral overexpression of HTRA1 promotes osteogenic differentiation of MSCs, while knockdown suppresses it. | **+0,68** | (27–31) |
| *OGT* | OGT | *Glycosyltransferase that catalyzes the addition of a single N-acetylglucosamine in O-glycosidic linkage to serine or threonine residues*  ○ Increased O-GlcNAc glycosylation is observed during the early stage of osteogenic differentiation in mouse pre-osteoblasts MC3T3-E1 cells.  ○ OGT inhibition by ST060266 reduces the expression levels of osteogenic genes and impairs mineralization. | **+0,68** | (32) |
| *APOD* | Apolipoprotein D | *Glycoprotein with antioxidant function*  ○ Osteogenic induction increases Apolipoprotein D expression levels.  ○ Apolipoprotein D addition to the culture medium enhances osteogenesis. | **+3,8** | (33,34) |
|  |  |  |  |  |
| *CRTAP*  *P3H1* | CRTAP  LEPRE1 | *Endoplasmic reticulum proteins involved in the biosynthesis and transport of type I collagen. Mutations in any of these proteins are associated with osteogenesis imperfecta or related disorders.*  *○* LEPRE1/CRTAP/ cyclophilin B complex mediate proline hydroxylation of type I collagen chain\| | **+0,58**  **+0,82** | (35,36) |
| *PLOD1* | LH1 | *○* LH1, LH2, and LH3 complex is involved in the lysine hydroxylation of type I collagen chains. | **+0,39** |  |
| *FKBP10* | FKBP65 | *○ FKBP65* catalyzes the cis-trans isomerization of peptide bonds at proline residues, acting as a chaperone for type I procollagen. It can bind to LH2 and enhance its enzymatic activity. | **+0,72** |  |
| *HSPA5* | BiP | *○* BiP acts as an endoplasmic reticulum stress sensor, and its expression increases in response to elevated collagen synthesis. It also contributes to the assembly of the FKBP65–LH2 complex, which is involved in the hydroxylation of the procollagen triple helix. | **+0,57** |  |
| *SERPINH1* | HSP47 | ○ HSP47 assists in the stabilization of the procollagen triple helix and its transport through the endoplasmic reticulum. | **+0,34** |  |
| *PDIA4* | Erp72 | ○ Erp72 is involved in oxidative protein folding through the formation, breakage, and rearrangement of disulfide bonds. | **+0,4** |  |
| *SEC23A*  *SEC24D*  *MIA2* | SEC23A  SEC24D  cTAGE5 | ○ SEC23A, SEC24D, and cTAGE5 are components of the COPII vesicle protein complex responsible for the anterograde transport of the procollagen triple helix from the endoplasmic reticulum to the Golgi apparatus. | **+0,25 +0,53**  **+0,41** |  |
| *ATP2A2* | SERCA2 | ○ Catalyzes the hydrolysis of ATP coupled with the calcium import into the sarcoplasmic reticulum lumen. Calcium is essential for collagen trimerization and proper folding. | **+0,39** |  |
| *PAPSS2* | 3'-Phosphoadenosine 5'-Phosphosulfate Synthase 2 | *Involved in the sulfation of ECM proteins.*  *○* PAPSS2 expression increases during osteogenesis of mouse pre-osteoblasts MC3T3-E1 cells.  ○ Reduced PAPSS2 levels attenuate TNAP activity and mineralization. | **+0,49** | (37) |

**TABLE S1**. **List of significantly regulated proteins in 14-3-3γ knockdown hASCs, treated with osteogenic differentiation medium (ODM).** Only significantly regulated (p < 0.05) proteins related to osteogenesis were included, organized according to their functional relevance. Proteins were selected based on a threshold of Log₂ fold change larger than 0.2 (upregulated) or smaller than -0.2 (downregulated). For each protein, the corresponding gene name, its reported involvement in osteogenesis, the Log₂ fold change (blue, downregulated; red, upregulated proteins), and the supporting bibliographic references are provided.

**Table S2**

| GENE | PROTEIN | MOLECULAR FUNCTION | REFERENCE |
| --- | --- | --- | --- |
| *ALPL* | Tissue Non-Specific Alkaline Phosphatase | *Plays an essential role in mineralization via inorganic pyrophosphate hydrolysis*  ○ TNAP contributes to AMPK activation, thereby promoting RUNX2 phosphorylation and its transcriptional activity.  ○ TNAP activity influences the expression of Runx2 and Sp7, as well as mature osteoblast and osteocyte markers like Bglap2 and Dmp1.  ○ TNAP is involved in mitochondrial function, ATP production, and the proliferation of bone progenitor cells. | (38–41) |
| *COL1A1*  *COL1A2* | Collagen Type I Alpha 1 Chain  Collagen Type I Alpha 2 Chain | *Both COL1A1 and COL1A2 encode the α-chains of type I collagen, the main organic component of bone matrix.*  ○ Type I collagen synthesis occurs early in pre-osteoblast cultures, and as differentiation progresses, the rate of synthesis declines while secretion and collagen fibrils formation increase.  ○ Disruption of their expression impairs bone formation and underlies diseases like osteogenesis imperfecta. | (15,35,36,42) |
| *CTSK* | Cathepsin K | *Cysteine protease highly expressed in osteoclasts, where it degrades type I collagen and other bone matrix proteins*  ○ Cathepsin K inhibition promotes osteogenic differentiation  ○ Bone marrow–derived mesenchymal stem cells express CTSK, but its levels decrease during the matrix mineralization stage. | (43,44) |
| *COL6A1*  *COL6A2*  *COL6A3* | Collagen Type VI Alpha 1 Chain  Collagen Type VI Alpha 2 Chain  Collagen Type VI Alpha 3 Chain | *The basic structural unit of collagen VI is a heterotrimer of the alpha1(VI), alpha2(VI), and alpha3(VI) chains*  ○ COL6A1, COL6A2 and COL6A3 show an increased expression during the osteogenic differentiation of periodontal ligament stem cells.  ○ Collagen type VI deficiency impairs bone formation | (15,45) |
| *COL12A1* | Collagen Type XII Alpha 1 Chain | *Alpha chain of type XII collagen*  ○ COL12A1 plays a critical role in late-stage osteogenic differentiation and contributes to cell–cell communication by regulating gap junction protein.  ○ COL12A1 silencing affects bone development showing reduced bone mass. | (46,47) |
| *ANXA2* | Annexin A2 | *Calcium-binding protein involved in the regulation of lipid rafts, inflammation, and fibrinolysis.*  ○ ANXA2 expression increases during osteogenic differentiation.  ○ ANXA2 deficiency impairs cell proliferation, alkaline phosphatase activity, and mineral deposition. | (15,48,49) |
| *MMP14* | Matrix Metallopeptidase 14 | *Extracellular protein involved in the breakdown of extracellular matrix*  ○ Deletion of MMP14 decreases ossification in mice osteoblast progenitors.  ○ MMP14 expression continuously declined during osteogenic differentiation of periodontal ligament stem cells. | (15,50,51) |
| *GREM1* | Gremlin-1 | *Secreted glycosylated protein*  ○ Gremlin-1 acts as a negative regulator of osteogenic differentiation by antagonizing BMP signaling.  ○ Overexpression of Gremlin 1 in hASC reduces TNAP activity, mineralization, and expression of osteogenic markers. | (15,52–54) |
| *FBN1* | Fibrillin-1 | *Extracellular matrix glycoprotein that assists in the assembly and stabilization of ECM complexes*  ○ FBN1 promotes osteogenic differentiation by enhancing the expression of osteogenic markers and increasing mineralization capacity.  ○ FBN1 expression rises during osteogenic induction, and overexpression of FBN1 leads to greater bone formation.  ○ FBN1 dysregulation impairs bone formation via TGF-β signaling | (15,55–57) |
| *PLOD2* | Lysyl Hydroxylase 2 | *Regulator of collagen cross-linking by hydroxylation of lysyl residues*  ○ PLOD2 expression is significantly upregulated during the mineralization stage of osteogenic differentiation.  ○ Loss of PLOD2 function leads to defective collagen cross-linking, resulting in skeletal abnormalities and bone fragility. | (35,58) |
| *CRYAB* | Alpha-Crystallin B Chain | *Molecular chaperone that acts holding proteins in large soluble aggregates*  ○ CRYAB expression is upregulated during osteogenic differentiation of bone marrow mesenchymal stem cells.  ○ CRYAB promotes osteogenic differentiation via FTH1 stabilization and ferroptosis suppression. | (59,60) |
| *POSTN* | Periostin | *Matricellular protein which mediates integrin and Wnt/β-catenin signaling*  ○ Periostin promotes osteogenic differentiation via Wnt/β-catenin and integrin pathways.  ○ Periostin protects osteogenic potential under inflammatory conditions. | (61–63) |
| *DCN*  *BGN* | Decorin  Biglycan | *Two most abundant small proteoglycans present in the bone matrix*  ○ Decorin is expressed earlier in newly differentiating osteoblasts, while biglycan expression increases as osteoblasts mature.  ○ Loss of BGN, but not DCN, reduced trabecular bone mass, with bone loss being more pronounced in double KO mice.  ○ Both are upregulated in newly differentiated osteoblasts before matrix mineralization, suggesting roles in early bone formation  ○ DCN promoted the osteoblastic differentiation of Periodontal Ligament Stem Cells, but it did not affect the osteoblastic differentiation of Saos2 preosteoblasts.  ○ DCN inhibits matrix mineralization of MC3T3-E1 preosteoblasts *in vitro.* | (64–68) |
| *FMOD* | Fibromodulin | *Extracellular matrix proteoglycan*  ○ Fibromodulin enhances osteogenesis by inhibiting the TGF-β/SMAD signaling pathway.  ○ FMOD deficient mice display impaired collagen fibrillogenesis and matrix mineralization. | (69,70) |
| *THBS1* | Thrombospondin 1 | *Glycoprotein that mediates cell-to-cell and cell-to-matrix interactions*  ○ Thrombospondin 1 inhibits osteogenic differentiation of human mesenchymal stem cells primarily by activating latent TGF-β  ○ High levels of Thrombospondin 1 inhibit pericytes mineralization.  ○ THBS1 KO mice show increased bone mass and increased cortical bone size and thickness | (71–73) |

**TABLE S2**.  **List of not significantly regulated proteins in 14-3-3γ knockdown hASCs, treated with osteogenic differentiation medium (ODM).**  Only not significantly regulated (p > 0.05) proteins related to osteogenesis were included, organized according to their functional relevance. For each protein, the corresponding gene name, its reported involvement in osteogenesis, and the supporting bibliographic references are provided.

**Figure S4**

**
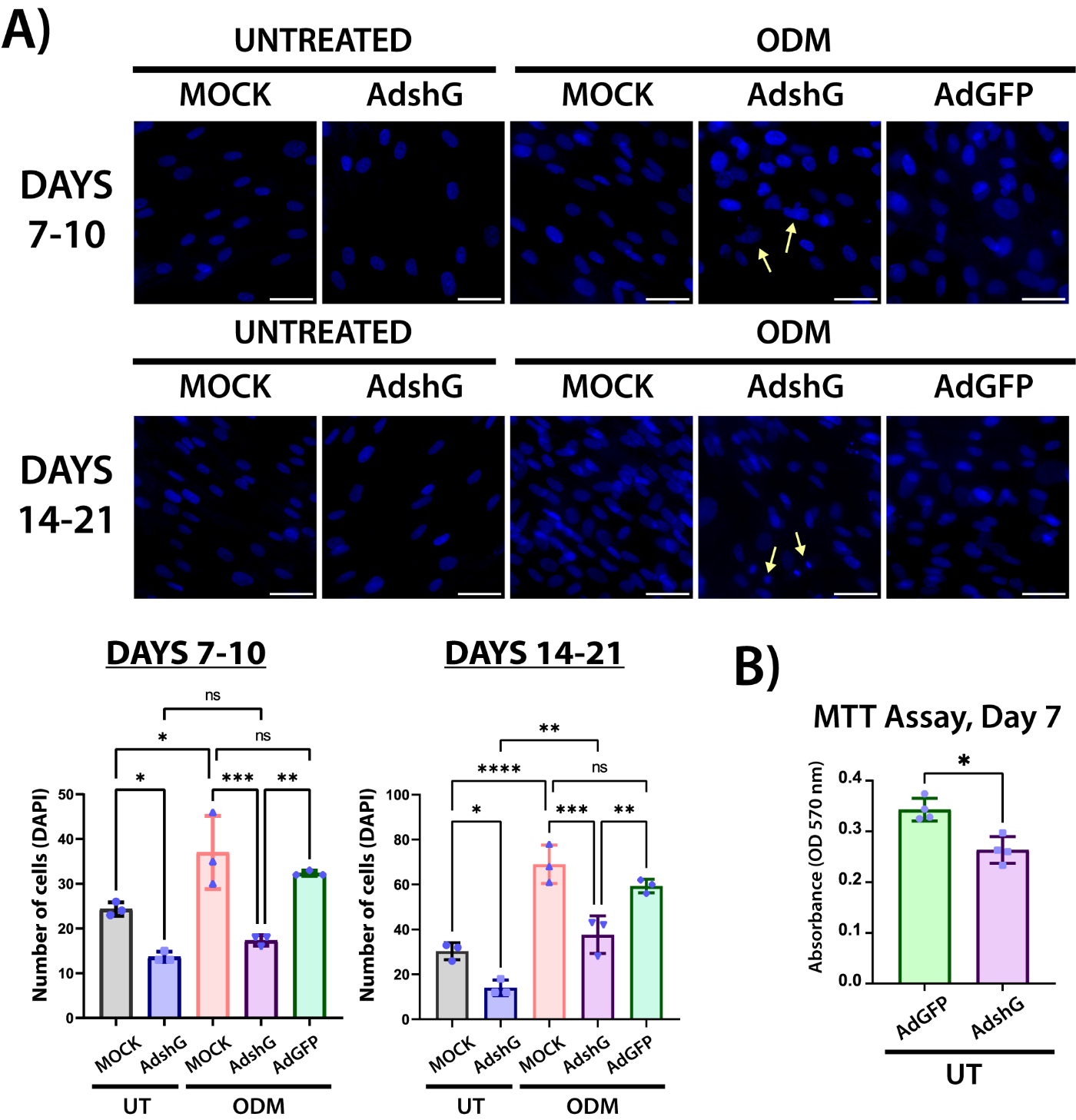
**

**Figure S4. Proliferation analysis following 14-3-3γ knockdown in hASCs. A)** Quantification of total cell number per visual field (DAPI-stained nuclei, 60× magnification) at days 7–10 and 14–21 of hASCs osteogenic differentiation. Representative images are shown for untreated: MOCK, Ad-shG-infected, and ODM-treated: MOCK, Ad-shG-, and Ad-GFP-infected hASCs. Yellow arrows indicate apoptotic cells according to changes in nuclear morphology (nuclear fragmentation and chromatin condensation). Nuclei numbers were assessed using Fiji-Imagej2 open-source software as previously described (74). Bar graphs (left-bottom) display the mean number of nuclei/field counted from three randomly selected fields per condition. Data are expressed as mean ± SD from three independent experiments (n = 3). Statistical differences were assessed by one-way ANOVA followed by Tukey’s post hoc test. *p < 0.05, **p < 0.01, ***p < 0.001, ****p < 0.0001; ns, not significant. Scale bars = 50 μm. **B)** MTT assay of Ad-shG- and Ad-GFP-infected hASCs grown in complete medium for 7 days. The MTT assay was performed following the methodology of a previous study (75). For each data point, the results are expressed as mean ± SD of four independent experiments. Statistical significance was determined using a paired Student’s *t* test. *p < 0.05.

**REFERENCES**

1. Liu X, Lian X, Liu X, Du Y, Zhu Y, Hu M, et al. Aldo-keto reductase family 1 member C1 regulates the osteogenic differentiation of human ASCs by targeting the progesterone receptor. *Stem Cell Res Ther*. **12**(1):383 (2021).

2. Rubin J, Schwartz Z, Boyan BD, Fan X, Case N, Sen B, et al. Caveolin-1 Knockout Mice Have Increased Bone Size and Stiffness. *J Bone Miner Res*. **22**(9):1408–18 (2007).

3. Baker N, Zhang G, You Y, Tuan RS. Caveolin‐1 regulates proliferation and osteogenic differentiation of human mesenchymal stem cells. *J Cell Biochem*. **113**(12):3773–87 (2012).

4. Baker N, Sohn J, Tuan RS. Promotion of human mesenchymal stem cell osteogenesis by PI3-kinase/Akt signaling, and the influence of caveolin-1/cholesterol homeostasis. *Stem Cell Res Ther*. **6**:238 (2015).

5. Su N, Chen M, Chen S, Li C, Xie Y, Zhu Y, et al. Overexpression of H1 calponin in osteoblast lineage cells leads to a decrease in bone mass by disrupting osteoblast function and promoting osteoclast formation. *J Bone Miner Res*. **28**(3):660–71 (2013).

6. Yoshikawa H, Taniguchi S, Yamamura H, Mori S, Sugimoto M, Miyado K, et al. Mice lacking smooth muscle calponin display increased bone formation that is associated with enhancement of bone morphogenetic protein responses. *Genes to Cells*. **3**(10):685–95 (1998).

7. Parente R, Sobacchi C, Bottazzi B, Mantovani A, Grčevic D, Inforzato A. The Long Pentraxin PTX3 in Bone Homeostasis and Pathology. *Front Immunol*. **10** (2019).

8. Zhang C, Hong FF, Wang CC, Li L, Chen JL, Liu F, et al. TRIB3 inhibits proliferation and promotes osteogenesis in hBMSCs by regulating the ERK1/2 signaling pathway. *Sci Rep*. **7**(1):10342 (2017).

9. Kim JM, Yang YS, Hong J, Chaugule S, Chun H, van der Meulen MC, et al. Biphasic regulation of osteoblast development via the ERK MAPK–mTOR pathway. *Elife*. **11** (2022).

10. Schindeler A, Little DG. Ras-MAPK Signaling in Osteogenic Differentiation: Friend or Foe? *J Bone Miner Res*. **21**(9):1331–8 (2006).

11. Arponen M, Jalava N, Widjaja N, Ivaska KK. Glucose transporters GLUT1, GLUT3, and GLUT4 have different effects on osteoblast proliferation and metabolism. *Front Physiol*. **13** (2022).

12. Liu L, Xie H, Zhao S, Huang X. The GLUT1–mTORC1 axis affects odontogenic differentiation of human dental pulp stem cells. *Tissue Cell*. **76**:101766 (2022).

13. Wei J, Shimazu J, Makinistoglu MP, Maurizi A, Kajimura D, Zong H, et al. Glucose Uptake and Runx2 Synergize to Orchestrate Osteoblast Differentiation and Bone Formation. *Cell*. **161**(7):1576–91 (2015).

14. Kuroda M, Ueda K, Kioka N. Vinexin family (SORBS) proteins regulate mechanotransduction in mesenchymal stem cells. *Sci Rep*. **8**(1):11581 (2018).

15. Li J, Wang Z, Huang X, Wang Z, Chen Z, Wang R, et al. Dynamic proteomic profiling of human periodontal ligament stem cells during osteogenic differentiation. *Stem Cell Res Ther*. **12**(1):1–16 (2021).

16. Qiu W, Sun Q, Li N, Chen Z, Wu H, Chen Z, et al. Superoxide dismutase 2 scavenges ROS to promote osteogenic differentiation of human periodontal ligament stem cells by regulating Smad3 in alveolar bone‐defective rats. *J Periodontol*. (2023).

17. Gao J, Feng Z, Wang X, Zeng M, Liu J, Han S, et al. SIRT3/SOD2 maintains osteoblast differentiation and bone formation by regulating mitochondrial stress. *Cell Death Differ*. **25**(2):229–40 (2018).

18. Schoppa AM, Chen X, Ramge JM, Vikman A, Fischer V, Haffner-Luntzer M, et al. Osteoblast lineage Sod2 deficiency leads to an osteoporosis-like phenotype in mice. *Dis Model Mech*. **15**(5) (2022).

19. Jiang W, Xing C, Wang H, Wang W, Chen S, Ning L, et al. A Lox/CHOP‐10 crosstalk governs osteogenic and adipogenic cell fate by MSCs. *J Cell Mol Med*. **22**(10):5097–108 (2018).

20. Rosset EM, Bradshaw AD. SPARC/osteonectin in mineralized tissue. *Matrix Biol*. **52**–**54**:78–87 (2016).

21. Hatori T, Maeda T, Suzuki A, Takahashi K, Kato Y. SPARC is a decoy counterpart for c‑Fos and is associated with osteoblastic differentiation of bone marrow stromal cells by inhibiting adipogenesis. *Mol Med Rep*. **27**(2):50 (2023).

22. Zhu Y, Gu Y, Jiang C, Chen L. Osteonectin regulates the extracellular matrix mineralization of osteoblasts through P38 signaling pathway. *J Cell Physiol*. **235**(3):2220–31 (2020).

23. Delany AM, Amling M, Priemel M, Howe C, Baron R, Canalis E. Osteopenia and decreased bone formation in osteonectin-deficient mice. *J Clin Invest*. **105**(7):915–23 (2000).

24. Liu F, Wu M, Wu X, Chen D, Xie M, Pan H. TGM2 accelerates migration and differentiation of BMSCs by activating Wnt/β-catenin signaling. *J Orthop Surg Res*. **18**(1):168 (2023).

25. Yang Z, Zhang X, Zhuo F, Liu T, Luo Q, Zheng Y, et al. Allosteric Activation of Transglutaminase 2 via Inducing an “Open” Conformation for Osteoblast Differentiation. *Adv Sci*. **10**(18) (2023).

26. Al-Jallad HF, Nakano Y, Chen JLY, McMillan E, Lefebvre C, Kaartinen MT. Transglutaminase activity regulates osteoblast differentiation and matrix mineralization in MC3T3-E1 osteoblast cultures. *Matrix Biol*. **25**(3):135–48 (2006).

27. Li R, Zhang Q. HtrA1 may regulate the osteogenic differentiation of human periodontal ligament cells by TGF-β1. *J Mol Histol*. **46**(2):137–44 (2015).

28. Tossetta G, Fantone S, Licini C, Marzioni D, Mattioli-Belmonte M. The multifaced role of HtrA1 in the development of joint and skeletal disorders. *Bone*. **157**:116350 (2022).

29. Tiaden AN, Breiden M, Mirsaidi A, Weber FA, Bahrenberg G, Glanz S, et al. Human Serine Protease HTRA1 Positively Regulates Osteogenesis of Human Bone Marrow-derived Mesenchymal Stem Cells and Mineralization of Differentiating Bone-forming Cells Through the Modulation of Extracellular Matrix Protein. *Stem Cells*. **30**(10):2271–82 (2012).

30. Hadfield KD, Rock CF, Inkson CA, Dallas SL, Sudre L, Wallis GA, et al. HtrA1 Inhibits Mineral Deposition by Osteoblasts. *J Biol Chem*. **283**(9):5928–38 (2008).

31. Filliat G, Mirsaidi A, Tiaden AN, Kuhn GA, Weber FE, Oka C, et al. Role of HTRA1 in bone formation and regeneration: In vitro and in vivo evaluation. *PLoS One*. **12**(7):e0181600 (2017).

32. Koyama T, Kamemura K. Global increase in O-linked N-acetylglucosamine modification promotes osteoblast differentiation. *Exp Cell Res*. **338**(2):194–202 (2015).

33. Yu RH, Zhang X yang, Xu W, Li Z kun, Zhu X dong. Apolipoprotein D alleviates glucocorticoid-induced osteogenesis suppression in bone marrow mesenchymal stem cells via the PI3K/Akt pathway. *J Orthop Surg Res*. **15**(1):307 (2020).

34. Martineau C, Najyb O, Signor C, Rassart É, Moreau R. Apolipoprotein D deficiency is associated to high bone turnover, low bone mass and impaired osteoblastic function in aged female mice. *Metabolism*. **65**(9):1247–58 (2016).

35. Claeys L, Storoni S, Eekhoff M, Elting M, Wisse L, Pals G, et al. Collagen transport and related pathways in Osteogenesis Imperfecta. *Hum Genet*. **140**(8):1121–41 (2021).

36. Malhotra V, Erlmann P. The Pathway of Collagen Secretion. *Annu Rev Cell Dev Biol*. **31**(1):109–24 (2015).

37. Wang W, Li F, Wang K, Cheng B, Guo X. PAPSS2 Promotes Alkaline Phosphates Activity and Mineralization of Osteoblastic MC3T3-E1 Cells by Crosstalk and Smads Signal Pathways. *PLoS One*. **7**(8):e43475 (2012).

38. Bartlett CL, Cave EM, Crowther NJ, Ferris WF. A new perspective on the function of Tissue Non-Specific Alkaline Phosphatase: from bone mineralization to intra-cellular lipid accumulation. *Mol Cell Biochem*. **477**(8):2093–106 (2022).

39. Zhang Z, Nam HK, Crouch S, Hatch NE. Tissue Nonspecific Alkaline Phosphatase Function in Bone and Muscle Progenitor Cells: Control of Mitochondrial Respiration and ATP Production. *Int J Mol Sci*. **22**(3):1140 (2021).

40. Nakamura T, Nakamura-Takahashi A, Kasahara M, Yamaguchi A, Azuma T. Tissue-nonspecific alkaline phosphatase promotes the osteogenic differentiation of osteoprogenitor cells. *Biochem Biophys Res Commun*. **524**(3):702–9 (2020).

41. Briolay A, Bessueille L, Magne D. TNAP: A New Multitask Enzyme in Energy Metabolism. *Int J Mol Sci*. **22**(19):10470 (2021).

42. Mollentze J, Durandt C, Pepper MS. An In Vitro and In Vivo Comparison of Osteogenic Differentiation of Human Mesenchymal Stromal/Stem Cells. *Stem Cells Int*. **2021**:1–23 (2021).

43. Mandelin J, Hukkanen M, Li TF, Korhonen M, Liljeström M, Sillat T, et al. Human osteoblasts produce cathepsin K. *Bone*. **38**(6):769–77 (2006).

44. Zhang W, Dong Z, Li D, Li B, Liu Y, Zheng X, et al. Cathepsin K deficiency promotes alveolar bone regeneration by promoting jaw bone marrow mesenchymal stem cells proliferation and differentiation via glycolysis pathway. *Cell Prolif*. **54**(7) (2021).

45. Izu Y, Ezura Y, Mizoguchi F, Kawamata A, Nakamoto T, Nakashima K, et al. Type VI collagen deficiency induces osteopenia with distortion of osteoblastic cell morphology. *Tissue Cell*. **44**(1):1–6 (2012).

46. Izu Y, Sun M, Zwolanek D, Veit G, Williams V, Cha B, et al. Type XII collagen regulates osteoblast polarity and communication during bone formation. *J Cell Biol*. **193**(6):1115–30 (2011).

47. Shaik S, Martin EC, Hayes DJ, Gimble JM, Devireddy R V. Transcriptomic Profiling of Adipose Derived Stem Cells Undergoing Osteogenesis by RNA-Seq. *Sci Rep*. **9**(1):11800 (2019).

48. Klabklai P, Phetfong J, Tangporncharoen R, Isarankura-Na-Ayudhya C, Tawonsawatruk T, Supokawej A. Annexin A2 Improves the Osteogenic Differentiation of Mesenchymal Stem Cells Exposed to High-Glucose Conditions through Lessening the Senescence. *Int J Mol Sci*. **23**(20) (2022).

49. Gillette JM, Nielsen-Preiss SM. The role of annexin 2 in osteoblastic mineralization. *J Cell Sci*. **117**(3):441–9 (2004).

50. Karsdal MA, Andersen TA, Bonewald L, Christiansen C. Matrix Metalloproteinases (MMPs) Safeguard Osteoblasts from Apoptosis during Transdifferentiation into Osteocytes: MT1-MMP Maintains Osteocyte Viability. *DNA Cell Biol*. **23**(3):155–65 (2004).

51. Tang Y, Rowe RG, Botvinick EL, Kurup A, Putnam AJ, Seiki M, et al. MT1-MMP-dependent control of skeletal stem cell commitment via a β1-integrin/YAP/TAZ signaling axis. *Dev Cell*. **25**(4):402–16 (2013).

52. Hu K, Sun H, Gui B, Sui C. Gremlin-1 suppression increases BMP-2-induced osteogenesis of human mesenchymal stem cells. *Mol Med Rep*. **15**(4):2186–94 (2017).

53. Zhu Z, Xing X, Huang S, Tu Y. NAT10 Promotes Osteogenic Differentiation of Mesenchymal Stem Cells by Mediating N4-Acetylcytidine Modification of Gremlin 1. *Stem Cells Int*. **2021**:1–10 (2021).

54. Gazzerro E, Smerdel-Ramoya A, Zanotti S, Stadmeyer L, Durant D, Economides AN, et al. Conditional Deletion of Gremlin Causes a Transient Increase in Bone Formation and Bone Mass. *J Biol Chem*. **282**(43):31549–57 (2007).

55. Li L, Huang J, Liu Y. The extracellular matrix glycoprotein fibrillin-1 in health and disease. *Front Cell Dev Biol*. **11** (2024).

56. Yang H, Wang W, Liu H, Zhang C, Cao Y, Long L, et al. miR615-3p inhibited FBLN1 and osteogenic differentiation of umbilical cord mesenchymal stem cells by associated with YTHDF2 in a m6A-miRNA interaction manner. *Cell Prolif*. **57**(6):e13607 (2024).

57. Ning T, Ning C, Li S, Mo C, Liu Z, Wang H. Integrative proteomics and phosphoproteomics profiling on osteogenic differentiation of periodontal ligament stem cell. *Proteomics*. **22**(21) (2022).

58. Kang H, Strong AL, Sun Y, Guo L, Juan C, Bancroft AC, et al. The HIF-1α/PLOD2 axis integrates extracellular matrix organization and cell metabolism leading to aberrant musculoskeletal repair. *Bone Res*. **12**(1):17 (2024).

59. Kulterer B, Friedl G, Jandrositz A, Sanchez-Cabo F, Prokesch A, Paar C, et al. Gene expression profiling of human mesenchymal stem cells derived from bone marrow during expansion and osteoblast differentiation. *BMC Genomics*. **8**(1):70 (2007).

60. Tian B, Li X, Li W, Shi Z, He X, Wang S, et al. CRYAB suppresses ferroptosis and promotes osteogenic differentiation of human bone marrow stem cells via binding and stabilizing FTH1. *Aging (Albany NY)*. **16**(10):8965–79 (2024).

61. Wu Z, Dai W, Wang P, Zhang X, Tang Y, Liu L, et al. Periostin promotes migration, proliferation, and differentiation of human periodontal ligament mesenchymal stem cells. *Connect Tissue Res*. **59**(2):108–19 (2018).

62. Bonnet N, Garnero P, Ferrari S. Periostin action in bone. *Mol Cell Endocrinol*. **432**:75–82 (2016).

63. Liu S, Jin Z, Cao M, Hao D, Li C, Li D, et al. Periostin regulates osteogenesis of mesenchymal stem cells from ovariectomized rats through actions on the ILK/Akt/GSK-3β Axis. *Genet Mol Biol*. **44**(3) (2021).

64. Hua R, Han Y, Ni Q, Fajardo RJ, Iozzo R V., Ahmed R, et al. Pivotal roles of biglycan and decorin in regulating bone mass, water retention, and bone toughness. *Bone Res*. **13**(1):2 (2025).

65. Han B, Li Q, Wang C, Chandrasekaran P, Zhou Y, Qin L, et al. Differentiated activities of decorin and biglycan in the progression of post-traumatic osteoarthritis. *Osteoarthr Cartil*. **29**(8):1181–92 (2021).

66. Bianco P, Fisher LW, Young MF, Termine JD, Robey PG. Expression and localization of the two small proteoglycans biglycan and decorin in developing human skeletal and non-skeletal tissues. *J Histochem Cytochem*. **38**(11):1549–63 (1990).

67. Mochida Y, Duarte WR, Tanzawa H, Paschalis EP, Yamauchi M. Decorin modulates matrix mineralization in vitro. *Biochem Biophys Res Commun*. **305**(1):6–9 (2003).

68. Adachi O, Sugii H, Itoyama T, Fujino S, Kaneko H, Tomokiyo A, et al. Decorin Promotes Osteoblastic Differentiation of Human Periodontal Ligament Stem Cells. *Molecules*. **27**(23):8224 (2022).

69. Goldberg M, Septier D, Oldberg Å, Young MF, Ameye LG. Fibromodulin-deficient Mice Display Impaired Collagen Fibrillogenesis in Predentin as Well as Altered Dentin Mineralization and Enamel Formation. *J Histochem Cytochem*. **54**(5):525–37 (2006).

70. Wang K, Zhou M, Zhang Y, Jin Y, Xue Y, Mao D, et al. Fibromodulin facilitates the osteogenic effect of Masquelet’s induced membrane by inhibiting the TGF-β/SMAD signaling pathway. *Biomater Sci*. **12**(7):1898–913 (2024).

71. Bailey DuBose K, Zayzafoon M, Murphy-Ullrich JE. Thrombospondin-1 inhibits osteogenic differentiation of human mesenchymal stem cells through latent TGF-β activation. *Biochem Biophys Res Commun*. **422**(3):488–93 (2012).

72. Canfield AE, Sutton AB, Hoyland JA, Schor AM. Association of thrombospondin-1 with osteogenic differentiation of retinal pericytes in vitro. *J Cell Sci*. **109**(2):343–53 (1996).

73. Amend SR, Uluckan O, Hurchla M, Leib D, Veis Novack D, Silva M, et al. Thrombospondin-1 Regulates Bone Homeostasis Through Effects on Bone Matrix Integrity and Nitric Oxide Signaling in Osteoclasts. *J Bone Miner Res*. **30**(1):106–15 (2015).

74. Rivera L, Popov Pereira da Cunha MD, Sabbatella RJ, Del Veliz S, Abraham GA, Uhart M, et al. Nanofibrous GelMA-Based Scaffolds Support Human Adipose-Derived Mesenchymal Stem/Stromal Cell Adhesion, Viability, and Growth. *J Biomed Mater Res A*. **113**(5):e37914 (2025).

75. Frontini-López YR, Rivera L, Pocognoni CA, Roldán JS, Colombo MI, Uhart M, et al. Infectious Bursal Disease Virus Assembly Causes Endoplasmic Reticulum Stress and Lipid Droplet Accumulation. *Viruses*. **15**(6) (2023).
