# Supplementary material for: 14-3-3γ Knockdown promotes matrix mineralization in human mesenchymal stromal cells
Source: Cell Death Dis. 2026 Mar 27;17(1):415. doi: 10.1038/s41419-026-08540-4 (PMC13149861; doi:10.1038/s41419-026-08540-4)
Supplement: Supplementary file 2 — Related Manuscript File [file 41419_2026_8540_MOESM2_ESM.docx]

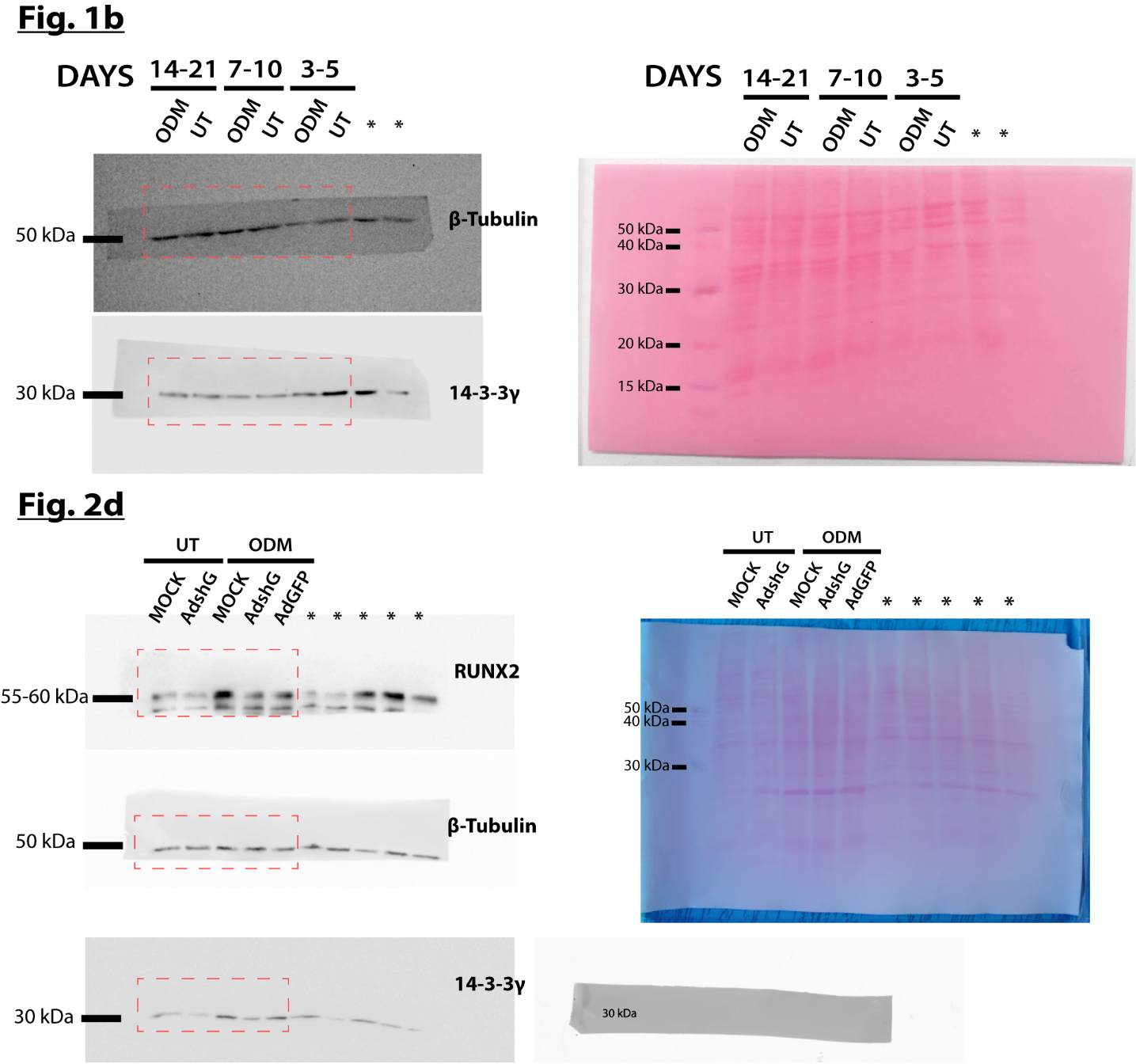


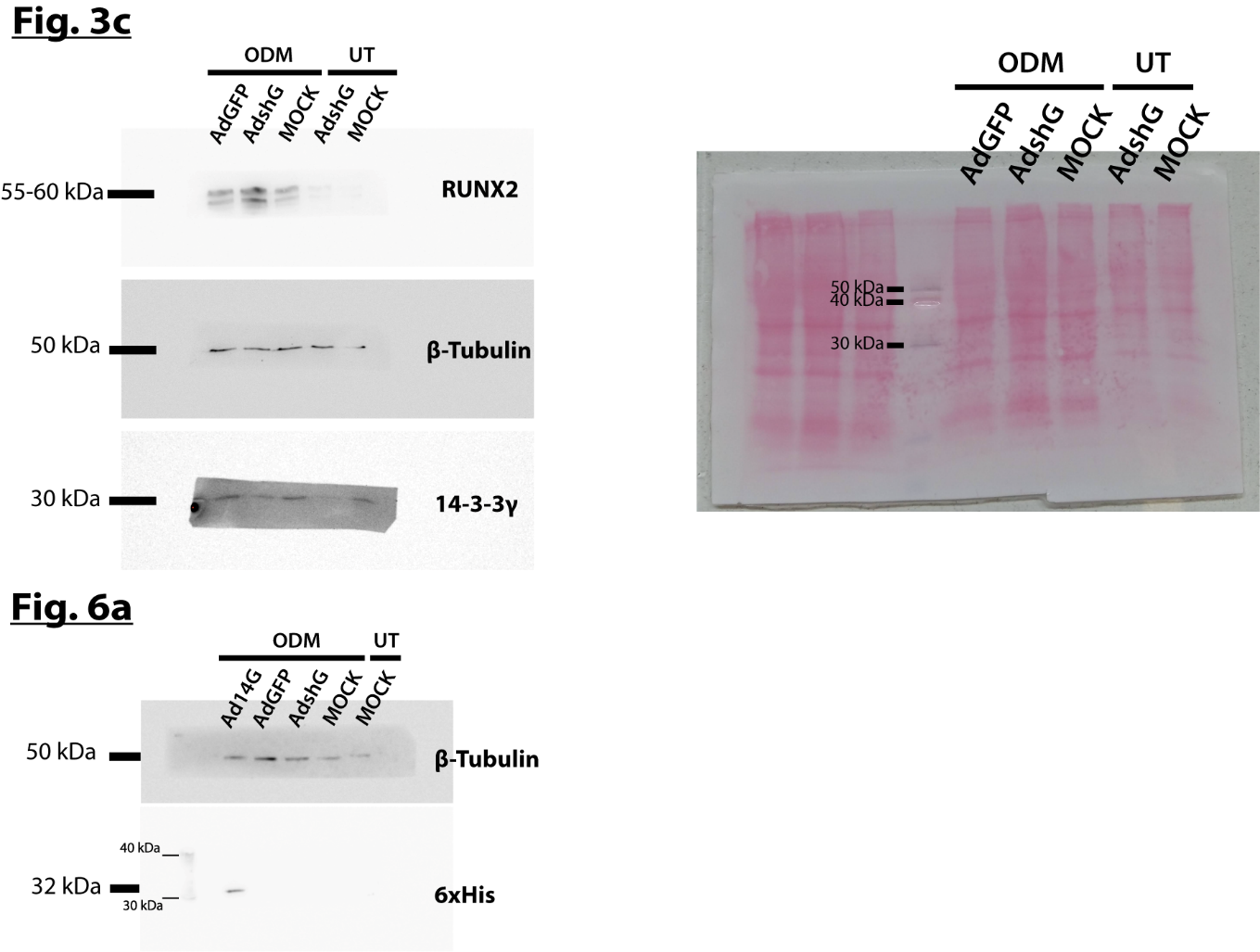


**Scans of the original western blots corresponding to Fig. 1b, Fig. 2d, Fig. 3c and Fig.6a.** In Fig. 1b and Fig. 2d, not all the samples were included in the present study, those are indicated with *. Bands presented and analyzed in those figures are highlighted with red boxes. Novex Sharp Pre-Stained Protein Standard (LC5800, Invitrogen) was used as a protein ladder.
